# Improved Configuration and LSPR Response of Platinum Nanoparticles via Enhanced Solid State Dewetting of In-Pt Bilayers

**DOI:** 10.1038/s41598-018-37849-0

**Published:** 2019-02-04

**Authors:** Sundar Kunwar, Mao Sui, Puran Pandey, Zenan Gu, Sanchaya Pandit, Jihoon Lee

**Affiliations:** Department of Electronic Engineering, College of Electronics and Information, Kwangwoon University, Nowon-gu Seoul, 01897 South Korea

## Abstract

Noble metallic nanoparticles (NPs) can exhibit valuable properties such as localized surface plasmon resonance (LSPR) and large surface to volume ratio, which can find various optoelectronic and catalytic applications. In this work, the improved configuration and uniformity of platinum (Pt) NPs are demonstrated by using a sacrificial indium (In) layer via the enhanced solid state dewetting of In-Pt bilayers on sapphire (0001). In a sharp contrast to the conventional dewetting of intrinsic Pt film, the introduction of In component can significantly enhance the global dewetting process and thus can result in the fabrication of well-defined Pt NPs with the improved uniformity. This can be due to the fact that In possess high diffusivity, low surface energy and low sublimation temperature. Upon annealing, the intermixing of In and Pt atoms can occur at the interface due to the inter-diffusion, which forms In-Pt alloy system. As a result, the overall diffusivity and dewetting degree of system can be significantly improved and this can produce more isolated, uniform and semispherical Pt NPs at much lower temperatures as compared to the pure Pt film dewetting. Conveniently, the In atoms preferentially can be removed from the NP matrix by the sublimation even at relatively low temperatures. These Pt NPs exhibit dynamic LSPR band in the UV-visible wavelength based on the excitation of dipolar, quadrupolar and higher order resonance modes. Specifically, the LSPR wavelength can be tuned between ~480 and 580 nm by the fabrication of small to large size Pt NPs with the distinct configuration and interparticle spacing. Furthermore, at a constant Pt thickness, the size, spacing and density of Pt NPs can be readily tuned by the control of In layer thickness. The introduction of sacrificial In component can enable an additional flexibility for the control of surface morphologies of metallic NPs with the low diffusivity materials.

## Introduction

Recently, a broad range of scientific and industrial applications including plasmonic, optoelectronic, energy, sensor and biomedical devices have been demonstrated based on the nanostructures of various noble metals^1–5^. The design of nanoparticles (NPs) in terms of their dimension, configuration, arrangement and uniformity has been the key aspect to achieve the desired and improved performances with the appropriate optical, catalytic, magnetic and electronic properties^6–9^. For instance, the power conversion efficiency of solar cell can be significantly improved by the incorporation of plasmonic Au NPs, which further allows to tune the absorption wavelength by controlling the particle size^10^. In particular, there has been intensive research interests on the localized surface plasmon resonance (LSPR) of metallic NP arrays and their interactions with the metallic, dielectric or semiconductor active layers^1,3,6^. The LSPR is generated by the collective oscillation of free electrons confined to the metallic NPs with the incident photons due to which the large electro-magnetic field enhancement, absorption and scattering can occur at the metal-dielectric or metal-air interfaces^11^. The peak position and bandwidth of LSPR can be determined by the size, shape and geometry of NPs, which makes it tunable conveniently by controlling those structural factors of plasmonic NPs^12^. Due to their strong interaction with the photon excitation, silver (Ag) and gold (Au) nanostructures have been widely adopted in various plasmonic applications, however, the Pt NPs have been rarely explored, which yet possess interesting plasmonic behaviors^13–15^. For instance, the small Pt NPs (<10 nm) have been utilized to enhance the photocurrent and thus the photocatalytic activity in various redox reaction due to the photon absorption by Pt NPs under visible light irradiation^2^. The fabrication of Pt NPs has been challenging especially through the physical vapor deposition and annealing as it requires high processing temperatures, typically over 800 °C. This is due to the fact that Pt atoms possess low diffusivity, which further limits the morphological and structural tunability^16,17^. In this regard, a detailed study to improve configuration, arrangement and uniformity of the Pt nanostructures and their optical properties can be of necessity to develop the plasmonic interfaces in the field of optoelectronics, sensing and catalysis^18–20^. In this paper, the improved plasmonic Pt nanostructures of various shape, size and configurations along with their optical characteristics are systematically demonstrated and studied using the enhanced thermal dewetting of continuous Pt layer deposited on a sacrificial In layer. In this approach, the indium layer serves as an enhancement factor for the global diffusivity of system through the inter-mixing of atoms. The In-Pt alloy system demonstrates much enhanced dewetting along with a preferential In atom sublimation. The dewetting characteristics are systematically controlled by the variation of film thickness, temperature and duration of annealing, which yields various Pt nanostructures such as nanoclusters, elongated NPs and semi-spherical NPs at relatively lower temperatures. Furthermore, the size and interparticle spacing of Pt NPs are readily tuned simply by the variation of sacrificial In layer thickness at an identical growth condition. The optical spectra analysis by reflectance, transmittance and extinction reveal the ample LSPR effect based on the size and configuration of Pt NPs.

## Results and Discussion

**Figure 1:**
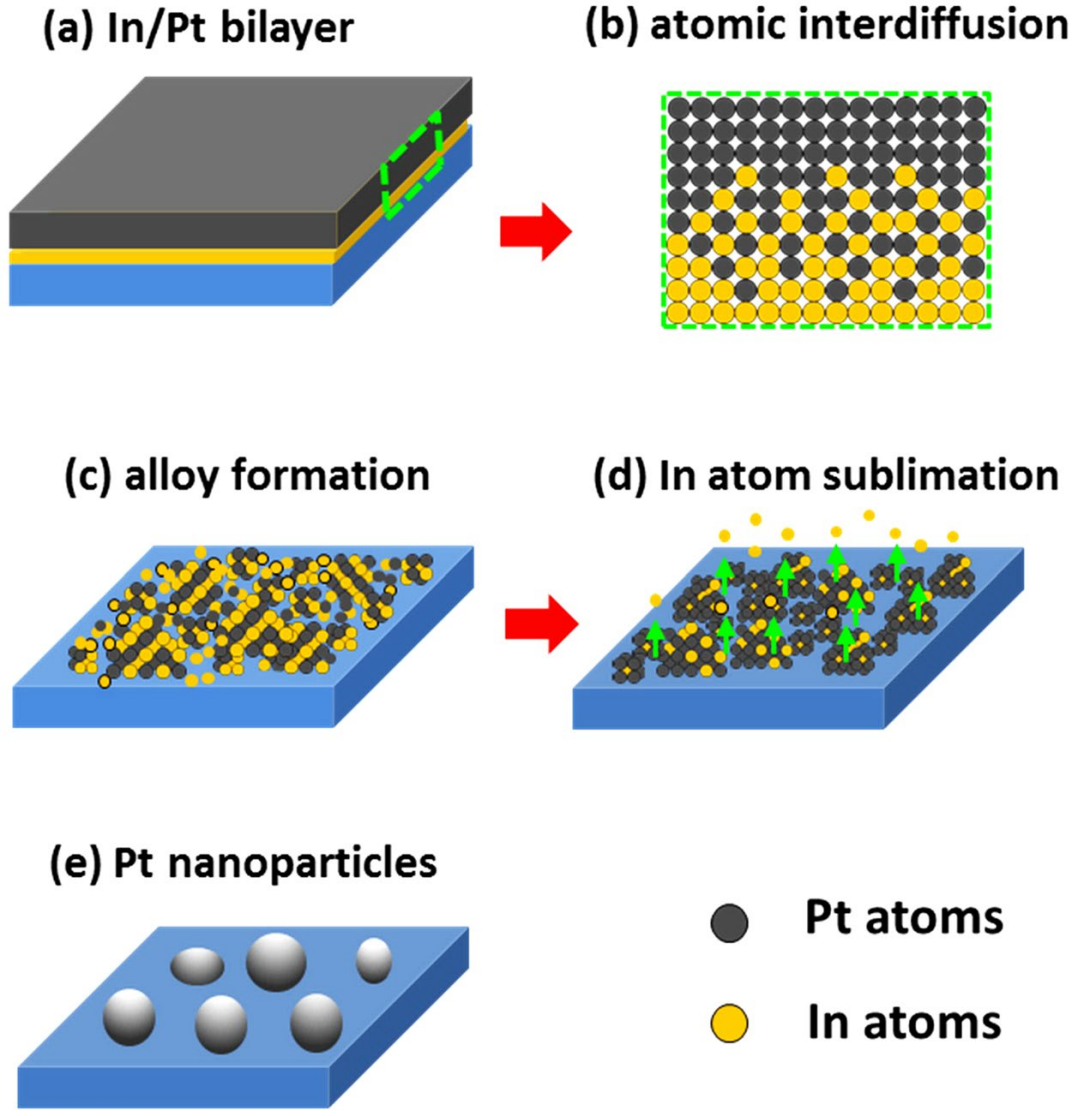
Schematic representation of fabrication process of self-assembled platinum (Pt) nanoparticles (NPs) on sapphire (0001) from the sputter deposited In/Pt bilayer. (**a**) Deposition of In and Pt bilayer with the Pt layer being on top. (**b**) Atomic inter-diffusion upon increased annealing temperature. (**c**) Alloy formation. (**d**) Sublimation of In atoms. (**e**) Formation of Pt NPs.

Figure 1 shows the overall fabrication process of Pt NPs on sapphire (0001) from the bilayer of In and Pt. The bilayer deposition schematic is shown in Fig. 1(a), in which the In is underlying and Pt is the top-layer, denoted as In/Pt bilayer. The evolution of Pt NPs from the sputtered In/Pt bilayers can be discussed based on the modified solid-state dewetting along with the concurrent effect of atomic diffusion and interdiffusion^16^. Generally, metallic films are not stable in the as-deposited state and when the appropriate thermal energy is applied, it can transform or dewet into isolated particles. This transformation is mainly driven by the total energy minimization of thermodynamic system^17^. The In layer was chosen to enhance overall dewetting process of Pt films because of its high diffusivity and high vapor pressure. In this case, the growth of Pt nanostructures by the dewetting of In/Pt bilayer can be explained in the following stages: (i) inter-diffusion of In and Pt atoms at the interface, (ii) formation of In-Pt binary system or alloying, (iv) partial dewetting of In-Pt alloy nanostructures and sublimation of In atoms and (v) evolution of nearly pure Pt nanostructures^21^. More specifically, the characteristics of sacrificial layer such as diffusivity, surface energy, melting temperature, thickness and interdiffusion with the over layer exhibit substantial effects on the overall dewetting of system^21,22^. In this case, the In possess a very low melting point (156.6 °C) and low surface energy whereas the top-layer Pt has a very high melting point (1768 °C) and much higher surface energy^23^. Therefore, the In atoms can be activated and begin to diffuse at relatively low temperature, which can incorporate into the Pt layer at the interface, giving rise to the interdiffusion process as illustrated in Fig. 1(b). Upon annealing at increased temperature, the formation of a lower temperature eutectic at the In-Pt interface can be expected and therefore atomic interdiffusion can be further enhanced, which result in the formation of In-Pt binary or alloyed system^24^. Therefore, the atomic interdiffusion and formation of In-Pt alloy can be the most important phenomenal aspect that enhances the overall diffusivity and drives the dewetting process. As the temperature increases further, the partial dewetting of alloy nanostructures can occur due to the enhanced surface diffusivity as illustrated in Fig. 1(c). As a result, eventually numerous voids, pits as well as some large In-Pt agglomerates can be expected to form on the surface. In the next stage of dewetting, the In atoms can be largely desorbed by the sublimation due to the fact that In has a low evaporation temperature as illustrated in Fig. 1(d), which leads to the evolution of nearly pure Pt nanostructures on sapphire. In contrast to the conventional dewetting of pure Pt films, the dewetting process can be significantly enhanced by using the sacrificial layer and effectively controlled, which will be discussed with different thickness of In/Pt bilayer films.

**Figure 2:**
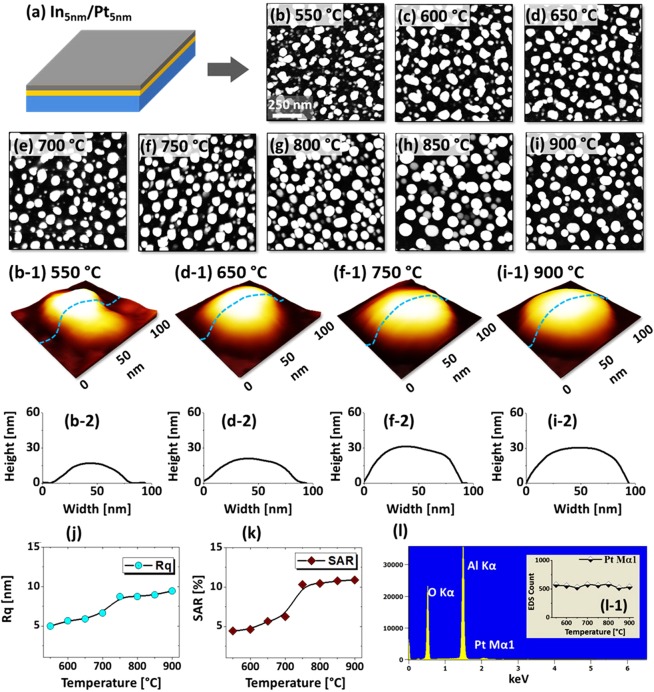
Evolution of semi-spherical Pt NPs by the control of temperature between 550 and 900 °C for 450 s with the bilayer of In_5nm_/Pt_5nm_. (**a**) Deposition schematic of In_5nm_/Pt_5nm_ bilayer. (b–i) Atomic force microscope (AFM) top views (1 × 1 μm^2^) of the Pt NPs at different temperatures as labelled. (b-1)–(i-1) 3D side-views of typical Pt NPs at specific temperatures. (b-2)–(i-2) Cross-sectional line-profiles of the corresponding 3D side-views. (**j**–**k**) Summary plots of RMS roughness (Rq) and surface area ratio (SAR). (**l**) Energy dispersive x-ray spectroscope (EDS) spectrum of a sample annealed at 550 °C. (l-1) Summary count plot of Pt Mα1 peak at 2.047 keV with respect to the temperature.

**Figure 3:**
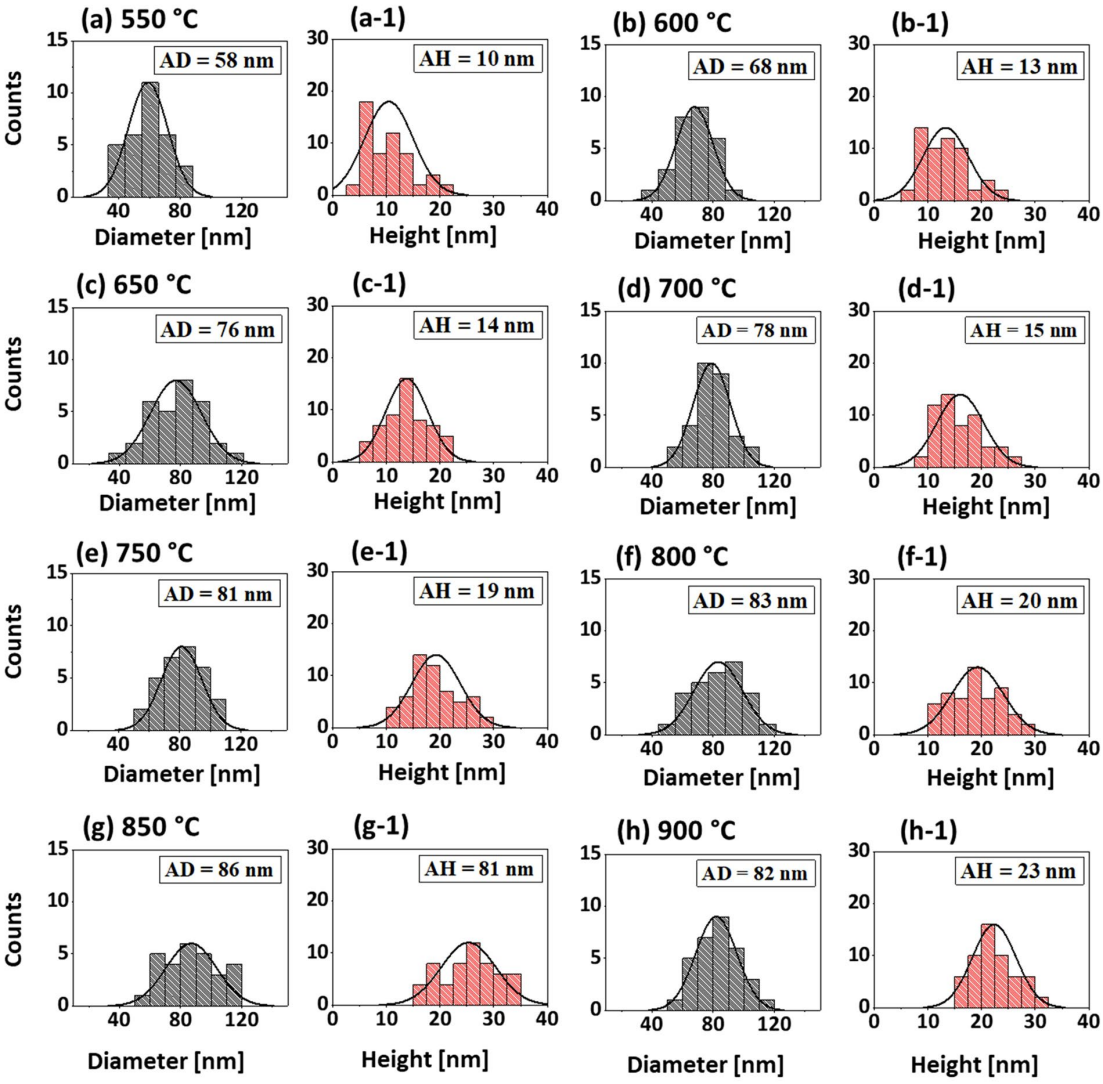
Size distribution histograms of Pt NPs fabricated with In_5nm_/Pt_5nm_ bilayer at different temperatures. (**a**–**h**) Diameter distribution histograms of Pt NPs in which the average diameter (AD) is indicated by the peak of normal distribution curves and labels. (a-1)–(h-1) Height distribution histograms of Pt NPs with average height (AD) as indicated by the peaks of normal distribution curve and labels. The AD and AH of Pt NPs were gradually increased with the temperatuure.

Figure 2 shows the evolution of Pt NPs from the In_5nm_/Pt_5nm_ bilayer based on the annealing between 550 and 900 °C. Initially, 5 nm thick In layer was deposited on sapphire (0001) and 5 nm thick Pt layer was added atop as shown in Fig. 2(a). Generally, the growth of surface nanostructures started with the formation of voids and irregular nanoclusters at low temperature and then isolated semi-spherical Pt NPs were gradually developed at increased temperature. In specific, isolated but slightly irregular Pt NPs were witnessed at 550 °C as shown in Fig. 2(b). The average size of Pt NPs was ~58 nm in diameter and ~10 nm in height as clearly displayed by the AFM side-views, corresponding line profile in Fig. 2(b-1),(b-2). Furthermore, the size distribution histograms of the Pt NPs are presented in Fig. 3(a),(a-1), which showed diameter and height variation at specific temperatures. As discussed, at the initial stage, the dewetting process can be initiated due to the enhanced diffusion of alloyed In-Pt atoms, which results in the pinholes/void nucleation at low energy sites and accumulation of atoms through the void rims. Consequently, the size, configuration and inter particle spacing were gradually improved along with the temperature, which can be attributed to the enhanced surface diffusion of atoms along with the sublimation of In atoms. Although a significant increase of NP height from ~15 to 25 nm and diameter from ~70 to 90 nm were observed at 650 °C with a notable reduction of density as displayed in Fig. 1(c,d), the NPs configuration remained somewhat elongated and irregular. The growth of the voids as well as interparticle spacing can be driven by the interfacial energy minimization between film and sapphire^25,26^. A better shape uniformity and enlarged spacing between NPs were realized when the temperature was increased between 750 and 900 °C as shown in Fig. 2(f–i). Meanwhile, randomly dispersed smaller NPs were attached to the larger NPs due to the coalescence, thereby causing lower density and larger size. The coalescence growth of isolated NPs can occur in order to gain the equilibrium or stable configuration^27^. The cross-sectional line profiles in Fig. 2(f-2)–(i-2) show the semi-spherical shape of Pt NPs, which can be generated due to the isotropic surface energy distribution of Pt NPs. As estimated, there was minor variation in diameter (~80 nm) but significant increment in height (~30 nm) of Pt NPs. In comparison with previous work, these results clearly demonstrated a significant improvement in size, uniformity and spacing of Pt NPs at similar Pt thickness (5 nm) and annealing temperature^16,17^. For instance, in the previous work of pure Pt films, the void nucleation was observed only above 700 °C and the evolution of NPs was hardly observed for the large variation of temperature up to 900 °C. The overall size distribution of Pt NPs is presented in Fig. 3, which also showed gradual increment in average height and diameter of NPs whereas improved uniformity. Therefore, the introduction of In component between the Pt film and sapphire demonstrated a great impact on the dewetting process. Furthermore, the growth behavior of Pt nanostructures is studied in terms of RMS roughness (Rq) and surface area ratio (SAR). Here, the Rq represents the average surface height whereas the SAR represents the 3D surface area of the nanostructures. The Rq is given as: $q=\sqrt{\frac{1}{n}\sum _{1}^{n}{Z}_{n}^{2}}$, where the *Zn* is the profile height at each pixel and the SAR is given as: $=\frac{{A}_{g}-{A}_{s}}{{A}_{g}}\times 100 \%$, where the *Ag* and *As* are geometric (2D) and surface area (3D) respectively. As summarized in Fig. 2(j,k), generally, both parameters were consistently increased along with the temperature due to the evolution of Pt nanostructures. Overall, the Rq and SAR were gradually increased from around 5 to 10 nm and 4 to 11% respectively while showing a large increase at 700 °C. In addition, the elemental analysis of each sample was performed by the EDS measurement. As shown in Fig. 2(l), three elemental peaks; O Kα, Al Kα and Pt Mα1 were commonly detected for all samples. In which, the O and Al peaks correspond to the substrate atoms and the Pt peak to nanostructure element. Due to the large desorption of In atoms, the EDS peak related to the In was not obtained even at 550 °C. However, minimal residual In atoms can be retained within the NPs which gradually desorb along with the dewetting at increased temperatures. It is also noted that the amount of Pt was constant throughout the temperature range as seen in summary plot in Fig. 2(l-1). This indicated that the total amount of Pt was identical regardless of different surface morphologies of Pt nanostructures.

**Figure 4:**
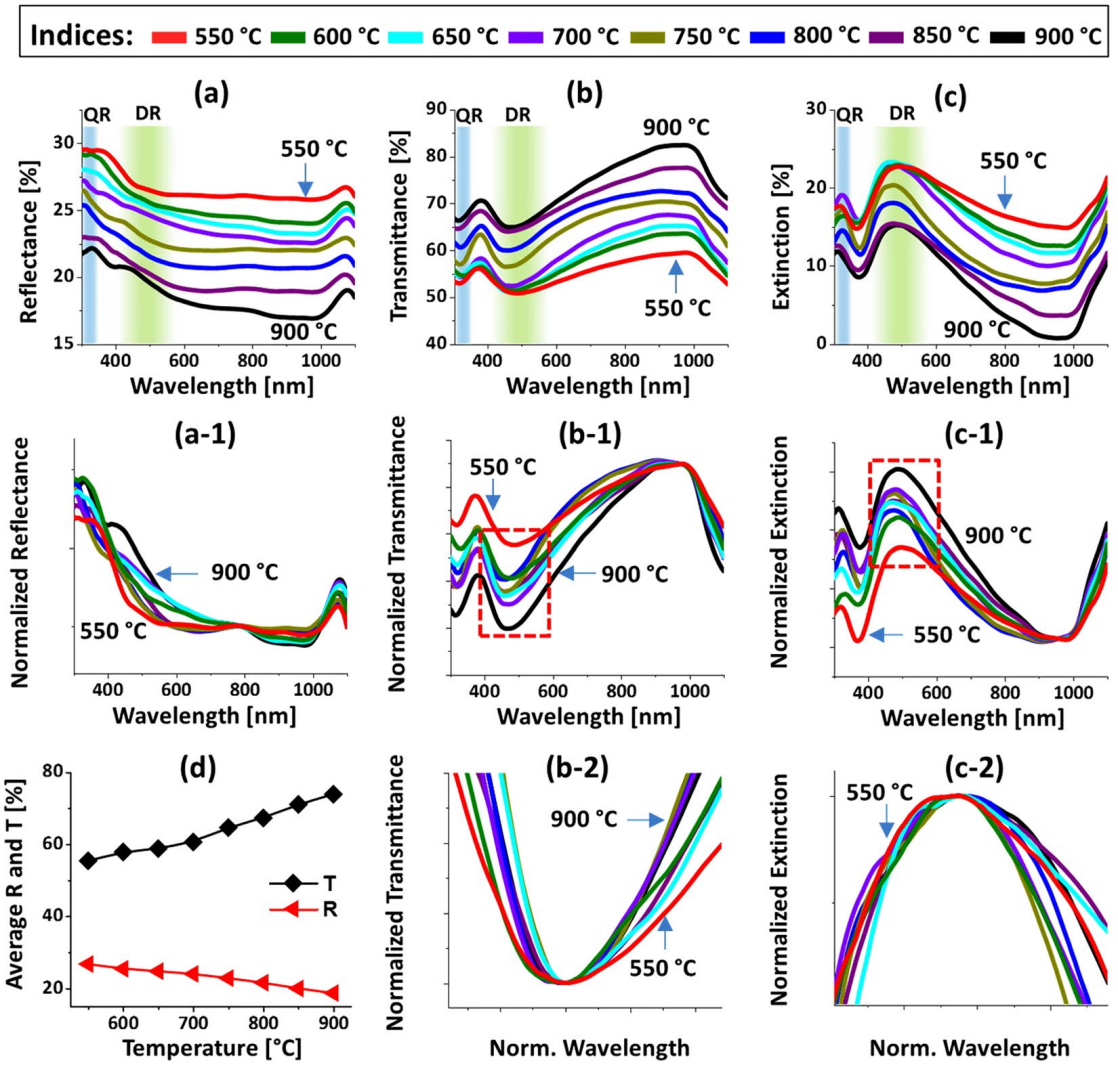
Optical properties of the semi-spherical Pt NPs fabricated with the In_5nm_/Pt_5nm_ bilayer between 550 and 900 °C for 450 s. (**a**) UV-VIS-NIR reflectance (R) spectra. (a-1) Normalized reflectance spectra. (**b**) Transmittance (T) spectra. (b-1) Normalized transmittance spectra. (b-2) Enlarged transmittance dip region, boxed region in (b-1). (**c**) Extinction spectra. (c-1) Normalized extinction spectra. (c-2) Zoomed in extinction peaks, boxed region in (c-1). Formation of quadrupolar (QR) and dipolar resonance (DR) modes are indicated by the color bands in (**a**–**c**). (**d**) Summary of average R and T.

Figure 4 displays the optical characterization of Pt NPs fabricated with the In_5nm_/Pt_5nm_ bilayer between 550 and 900 °C. The reflectance, transmittance and extinction spectra are shown in Fig. 4(a–c) respectively. In which, the reflectance and transmittance spectra were experimentally measured in a normal reflection and transmission mode and extinction spectra were extracted by the relation: extinction (%) = 100% − (reflectance + transmittance) %. Generally, the optical spectra were very sensitive to the morphology change of Pt NPs such that the formation of various resonance peaks and dips at specific wavelength regime and their intensity variation were observed. In specific, the reflectance spectra in Fig. 4(a) exhibited a downward slope in the visible to NIR region. In this set, generally the size of Pt NPs was below 100 and 30 nm in terms of diameter and height respectively. Although no obvious peaks or dips were resulted in the reflectance spectra, based on the given size of Pt NP formation of different (dipolar and quadrupolar) LSPR resonance mode can be anticipated in the UV and visible region^28^. However, the reflectance spectra exhibited a shoulder in the UV to visible region instead of the absorption dips, which can be likely due to the pronounced backscattering of UV and near visible wavelength by the dipolar NPs^29^. From the transmittance spectra in Fig. 4(b), it clearly showed a UV dip at ~320 nm and a wide visible dip at ~460 nm. The wavelength dependent dip formation in the transmittance spectra can be associated with the excitation of various LSPR resonance modes as indicated in Fig. 4(b)^28,30^. Specifically, the visible dips can be caused by the dipolar resonance mode whereas the UV dip by the quadrupolar resonance mode of Pt NPs^28,31^. At longer wavelength greater than 750 nm, while the reflectance was reduced, the transmittance was sharply increased making strong shoulder in the NIR region as shown in Fig. 4(a,b). Since the wavelength region was far from the LSPR excitation, the low reflectance and high transmittance in the NIR region may not be caused by the absorption and/or scattering enhancement. Instead, due to the reduction of refractive index mismatch between air and sapphire by the Pt NPs layer, the surface reflectance can be reduced whereas transmittance can be increased^32^. In terms of average magnitude, the reflectance was gradually decreased with respect to the temperature as shown in Fig. 4(d) due to the reduction of average surface coverage of Pt NPs. Whereas the average transmittance exhibited the increasing trend with temperature because of the increasing fraction of bare sapphire. The well-formed dips in the reflectance and/or transmittance spectra represents the absorption or scattering of the photons, which appear as peaks in extinction spectra as shown in Fig. 4(c). The positions of dipolar and quadrupolar resonance peaks were similar with the corresponding dip positions in the transmittance spectra. For the detail analysis of peak or dips trends along with the evolution of Pt NPs, the optical spectra were normalized and enlarged as shown in Fig. 4(a-1),(c-2). In specific, the reflectance spectra in Fig. 4(a-1) generally featured a shoulder in the UV to VIS region, which can be due to the backscattering effect along with the excitation of pronounced dipole resonance with small Pt NPs^29^. From the normalized transmittance spectra in Fig. 4(b-1), it can be seen that the UV-visible dips were gradually attenuated along with temperature, indicating the gradually enhanced absorption with the formation of larger and definite Pt NPs. Similarly, the extinction spectra in Fig. 4(c-1) had a strong peak in visible region (DR) and a minor peak in the UV region (QR) whose intensity were gradually increased with temperature, again likely due to the increased size and definite NP formation. In the case of low temperature samples, it consisted of a wide distribution of small and irregularly shaped Pt NPs and thus the LSPR peaks were normally broader. With increasing temperature, the Pt NPs became more uniform and semi-spherical, resulting in a narrowing of the LSPR band as shown in Figs. 4(b-2),(c-2)^32,33^. By comparing the optical spectra of Pt NPs on sapphire in the previous work^16^, the distinct and strong LSPR bands in the UV and VIS wavelengths were demonstrated because of the formation of definite, uniform and isolated Pt NPs. Furthermore, the LSPR bands were found to be more dynamic in correlation with the surface morphology change, which can be due to the enhanced dewetting by the sacrificial In component.

**Figure 5:**
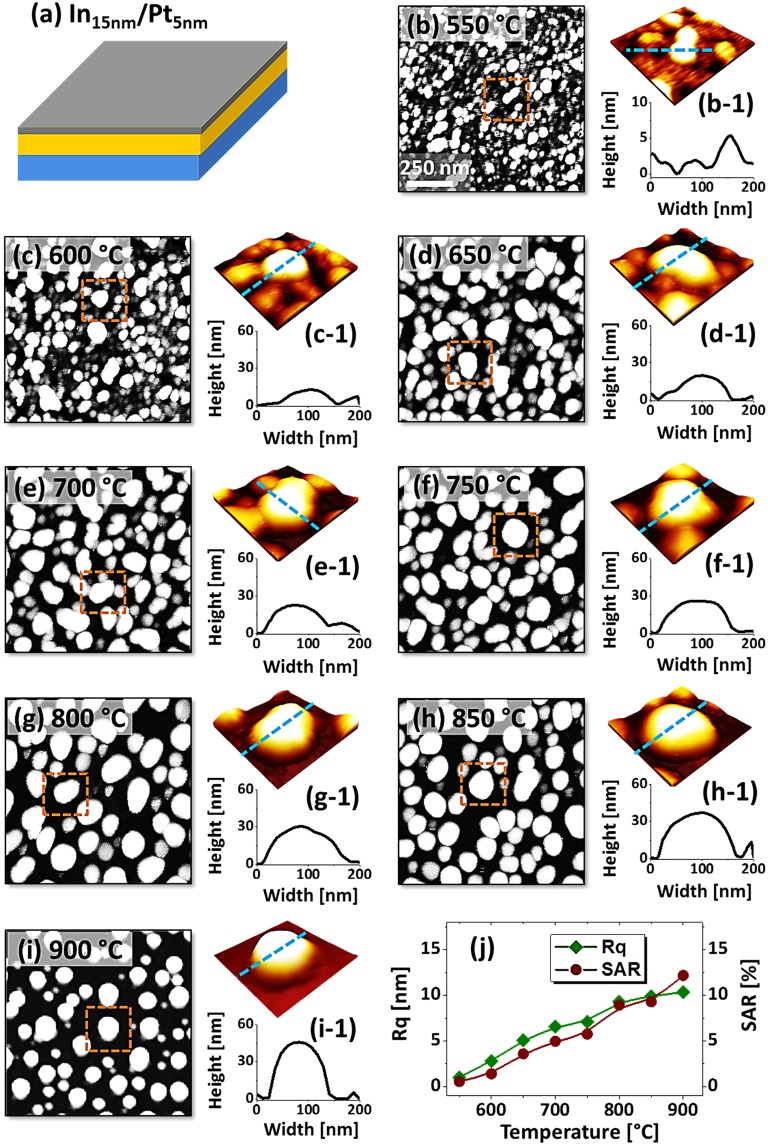
Fabrication of Pt NPs with the In_15nm_/Pt_5nm_ bilayer based on the annealing between 550 and 900 °C for 450 s. (**a**) Schematic of In_15nm_/Pt_5nm_ bilayer deposition. (**b**–**i**) AFM top-views of 1 × 1 μm^2^. (b-1)–(i-1) Typical Pt NPs at various temperature and corresponding line profiles. (**j**) Summary plots of Rq and SAR.

**Figure 6:**
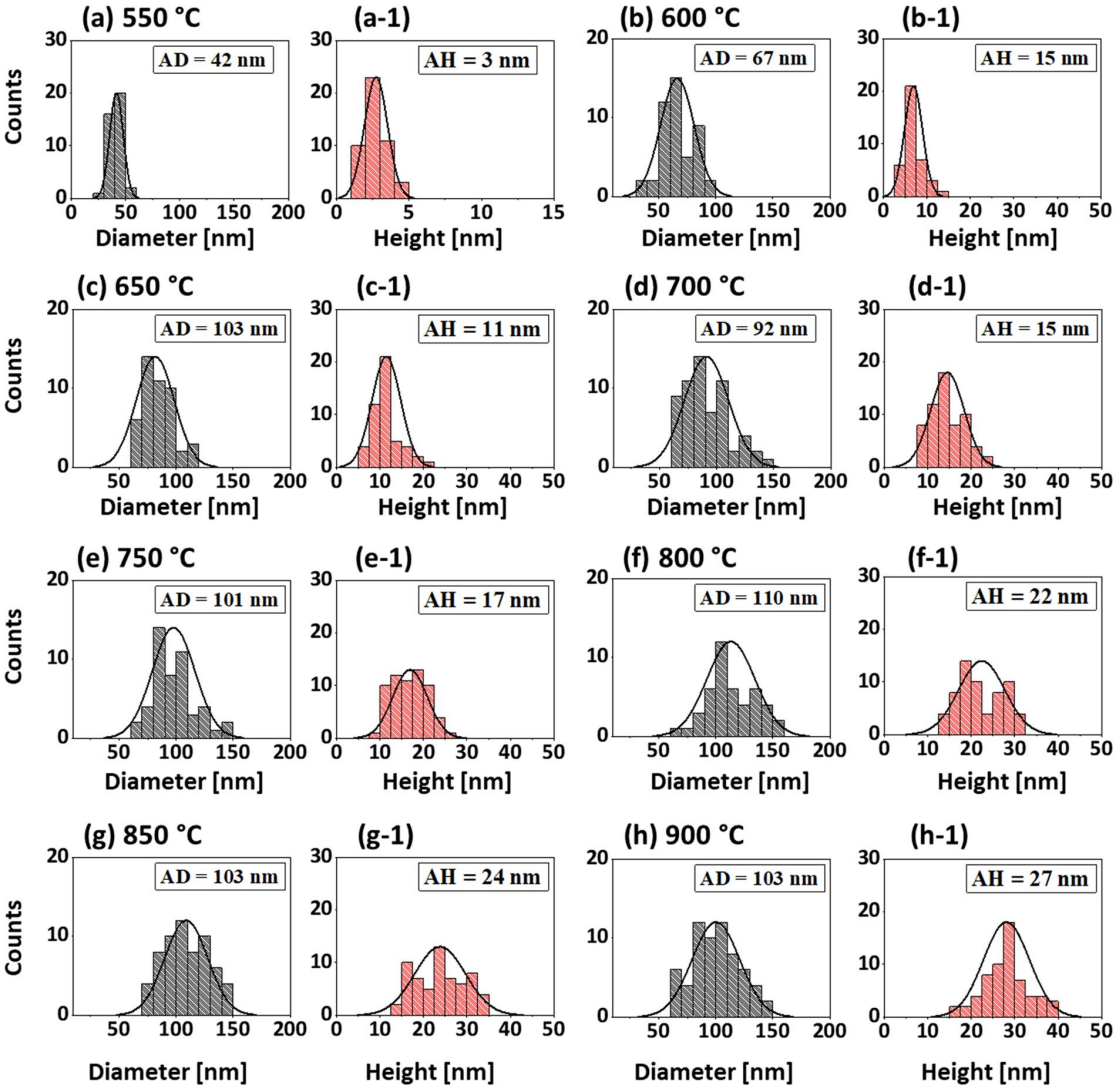
Size distribution histograms of Pt NPs fabricated with the In_15nm_/Pt_5nm_ bilayer at different temperatures. (**a**–**h**) Diameter distribution histograms of Pt NPs with the average diameter (AD) as indicated by the peak of normal distribution curves and labels. (a-1)–(h-1) (**a**–**h**) Height distribution histograms of Pt NPs with the average height (AD) as indicated by the peak of normal distribution curves and labels. The AD and AH of Pt NPs were gradually increased with the temperatuure.

**Figure 7:**
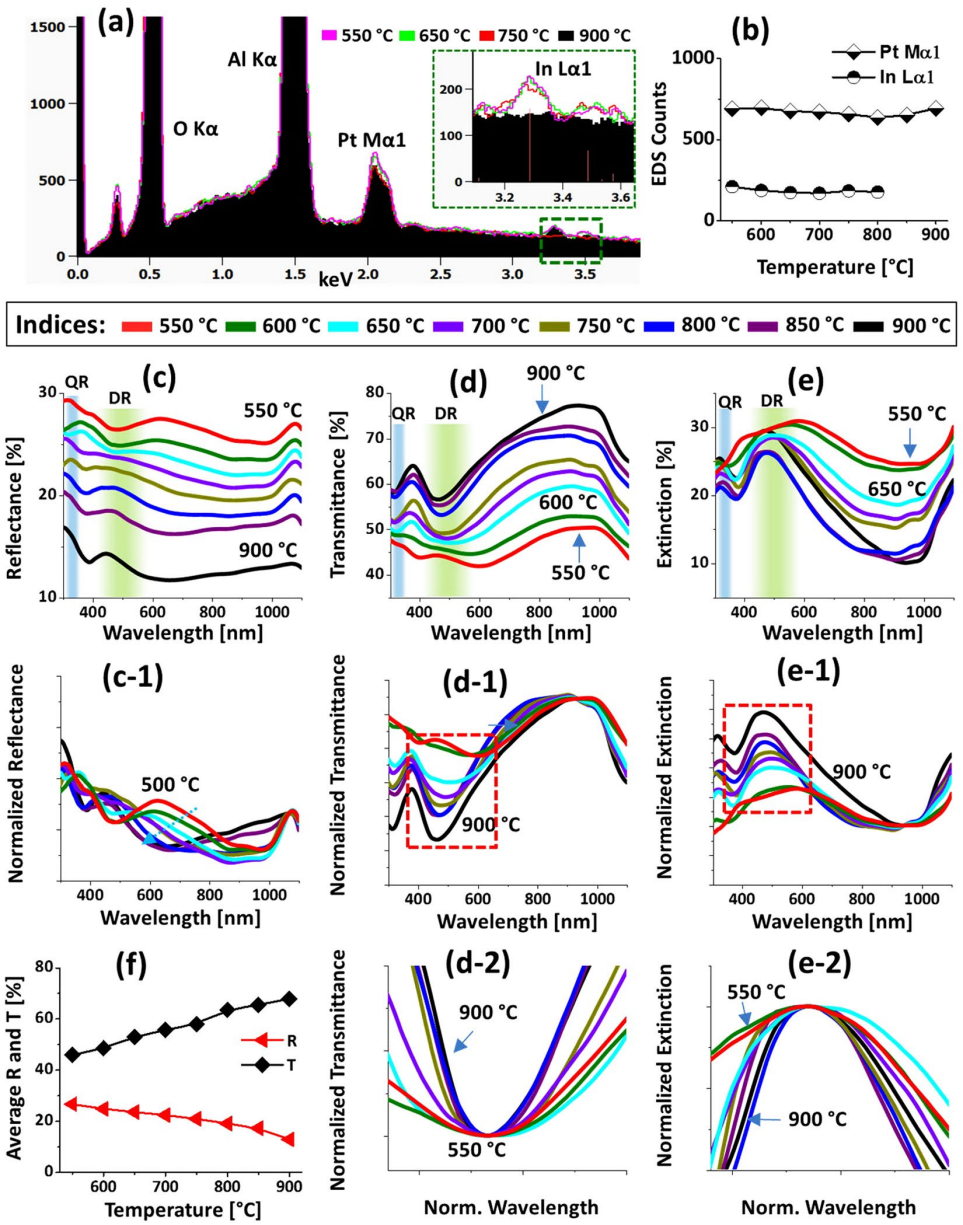
Elemental and optical properties of Pt NPs fabricated with the In_15nm_/Pt_5nm_ bilayer between 550 and 900 °C for 450 s. (**a**) EDS spectra of the samples annealed at various temperatures. Inset shows the corresponding In peaks. (**b**) Summary counts of Pt Mα1 peak at 2.047 keV and In Lα1 peak at 3.287 keV. (**c**) Reflectance (R) spectra. (c-1) Normalized reflectance spectra. (**d**) Transmittance (T) spectra. (d-1) Normalized transmittance spectra. (d-2) Enlarged dip region of transmittance spectra, boxed region in (d-1). (**e**) Extinction spectra. (e-1) Normalized extinction spectra. (e-2) Zoomed in extinction peaks, boxed region in (e-1). (**f**) Summary of average R and T.

Figure 5 shows the formation of various Pt NPs by the added thickness of In layer, the In_15nm_/Pt_5nm_ bilayer. This set of samples was prepared to clearly see the In thickness effect on the evolution of nanostructures at a constant Pt thickness and growth condition. As a sharp contrast to the previous set, the size, density and spatial arrangement of Pt NPs were drastically varied as seen in the AFM top-views. For instance, the Pt NPs obtained below 600 °C were slightly smaller and compact, however, the higher temperature samples showed much larger size and reduced areal density. Furthermore, during the dewetting of In/Pt bilayer, the transition at each annealing temperature was more dynamic and distinct, which can be related to the enhanced global diffusion by the increased thickness of In layer. The dewetting of thin film can be significantly altered by the variation of initial film thickness as well as the thickness of the constituent metal films. In this case, although the thickness of Pt layer was the same, the ratio of In component was also increased by 3 times, which resulted in a rapid and enhanced dewetting process. As the effectiveness of In atom interdiffusion and In-Pt alloying can be enhanced with the higher In thickness, the overall diffusivity of atomic system can also be enhanced^24^. Therefore, once the voids are nucleated, the dewetting process can advance much rapidly by the accumulation of diffusing atoms as well as sublimation of In atoms concurrently, which can result in the formation of well-defined Pt NPs even at much lower temperatures^33,34^. This set of samples showed a strong dependency of dewetting characteristic on the thickness of In film at constant Pt thickness^35^. In specific, the tiny NPs and voids were drastically developed into the large and compact wiggly NPs between 550 and 700 °C as shown in Fig. 5(b–d). The tiny NPs were highly dense at 550 °C, which can be likely due to the instantaneous nucleation of NPs due to the enhanced diffusivity of atoms in the In-Pt alloy system and concurrent sublimation of In atoms^33^. Along with the coalescence growth of adjacent NPs, the size of Pt NPs was gradually increased with the elongated-dome configuration whereas the density was correspondingly decreased^27^. The typical Pt NPs and corresponding line profiles are shown in Fig. 5(b-1)–(d-1). The NPs height and diameter were increased from ~5 to 20 nm and ~50 to 200 nm respectively. Comparing with the previous set, the average height was almost similar whereas the lateral diameter was increased about twice, indicating a preferential lateral growth. Subsequently, the large irregular NPs were gradually transformed into widely spaced semi-spherical NPs between 750 and 900 °C as shown in Fig. 5(f–i). The diameter of irregular large NPs was gradually contracted whereas the vertical height was enlarged along with the evolution of semi-spherical configuration, indicating a preferential vertical growth, which can be correlated to the surface and interface energy minimization by NPs to gain the thermal stability^36^. As seen from the cross-sectional line profiles, the average height was increased from ~30 to 45 nm whereas the diameter was slightly decreased form ~150 to 120 nm. It is also observed that the overall density of NPs was reduced in comparison with the previous set, which is accompanied by the increased size. The corresponding size distribution histograms of larger Pt NPs are presented in Fig. 6. Furthermore, the Rq and SAR values were increased more steeply such that the Rq and SAR were increased from ~3 to 10 nm and ~1 to 12% respectively as shown in Fig. 5(j). The elements present in the nanostructures were confirmed by the EDS spectra as shown in Fig. 7(a). As seen in the inset, the minor In Lα1 peaks were observed between 550 and 800 °C whereas it was completely vanished above 800 °C as shown in the summary plot in Fig. 7(b). This suggests that the small amount of In was still retained within the NPs up to 800 °C as three times higher In (15 nm) was used in this set and therefore, the dewetting process appears to be more dynamic even at high temperatures. It is also confirmed that the Pt deposition amount was constant for all samples in Fig. 7(b), which is similar to the previous set. The corresponding optical properties of this series of samples are demonstrated by the reflectance, transmittance and extinction in Fig. 7(c–e). Comparing with the previous set, the optical characteristics were obviously varied as the Pt NPs in this sets were bigger in terms of the average diameter, generally greater than 170 nm and widely spaced. Since the evolution of Pt NPs was more dynamic with the higher In component, the optical evolution was also clearly altered between the samples annealed from 550 to 900 °C. In specific, the reflectance spectra in Fig. 7(c) showed shoulders in the UV region at ~320 nm and in the visible region between 450 and 600 nm. As discussed, the LSPR resonance of Pt NPs can be significantly affected due to the backscattering, which may cause the shoulders in the UV and visible region of reflectance spectra^28^. For the low annealing temperature samples, the visible region shoulder was much wider, which became narrower with the increased temperature likely due to the improved uniformity of Pt NPs. From the transmittance spectra in Fig. 7(d), the quadrupolar and dipolar resonance dips were clearly observed in the UV and visible regions respectively. Regardless of distinct surface morphology of Pt NPs, the quadrupolar resonance was found to be consistent for all samples like the previous set. However, the dipolar resonance dips were significantly altered. For instance, the dipolar resonance dips were red shifted in comparison with the previous set. Furthermore, due to the wide size distribution of Pt NPs at low annealing temperature (550–600 °C), the dipolar resonance peak was broader and then it was significantly narrowed with the improved uniformity of size and shape at higher temperature^31^. The average reflectance and transmittance at each annealing temperature are summarize in Fig. 7(f), which showed gradually increasing trend of transmittance and decreasing reflectance based on the gradually reduced surface coverage of Pt NPs. Further evidence of the LSPR mode of Pt NPs is demonstrated by the extinction spectra in Fig. 7(e), which also suggested the quadrupolar and dipolar resonance peaks in the UV and visible region respectively^28,31^. The low extinction in the NIR region can be attributed to the high transmittance and low plasmonic resonance absorption. The normalized spectra are presented in Fig. 7(c-1)–(e-2) to demonstrate the LSPR trend along with the evolution of Pt NPs. As shown in Fig. 7(c-1), the normalized reflectance generally exhibited shoulders in UV and visible region and the visible region shoulder was gradually constructed towards shorter wavelength as the temperature increased. This can be attributed to the improved to the size and shape uniformity of Pt NPs as discussed. In Fig. 7(d-1), the UV and visible region dips were shown to extend deeper with the increasing temperature. This clearly suggested that the intensity of absorption by Pt NPs was gradually enhanced with the formation of definite, widely spaced and larger NPs. Consequently, the intensity of LSPR peaks were enhanced in the extinction spectra as displayed in Fig. 7(e-1). From the normalized spectra, it was found that the dipolar resonance peaks were more sensitive to the morphology change of Pt NPs than the quadrupolar resonance peaks. The uniformity improvement of Pt NPs along with the temperature substantially narrowed the LSPR band as shown in Fig. 7(d-2),(e-2). By comparing with the previous set, the narrowing effect was more significant in this set because of the further improvement of size and shape of Pt NPs with the enhanced diffusivity of the system.

**Figure 8:**
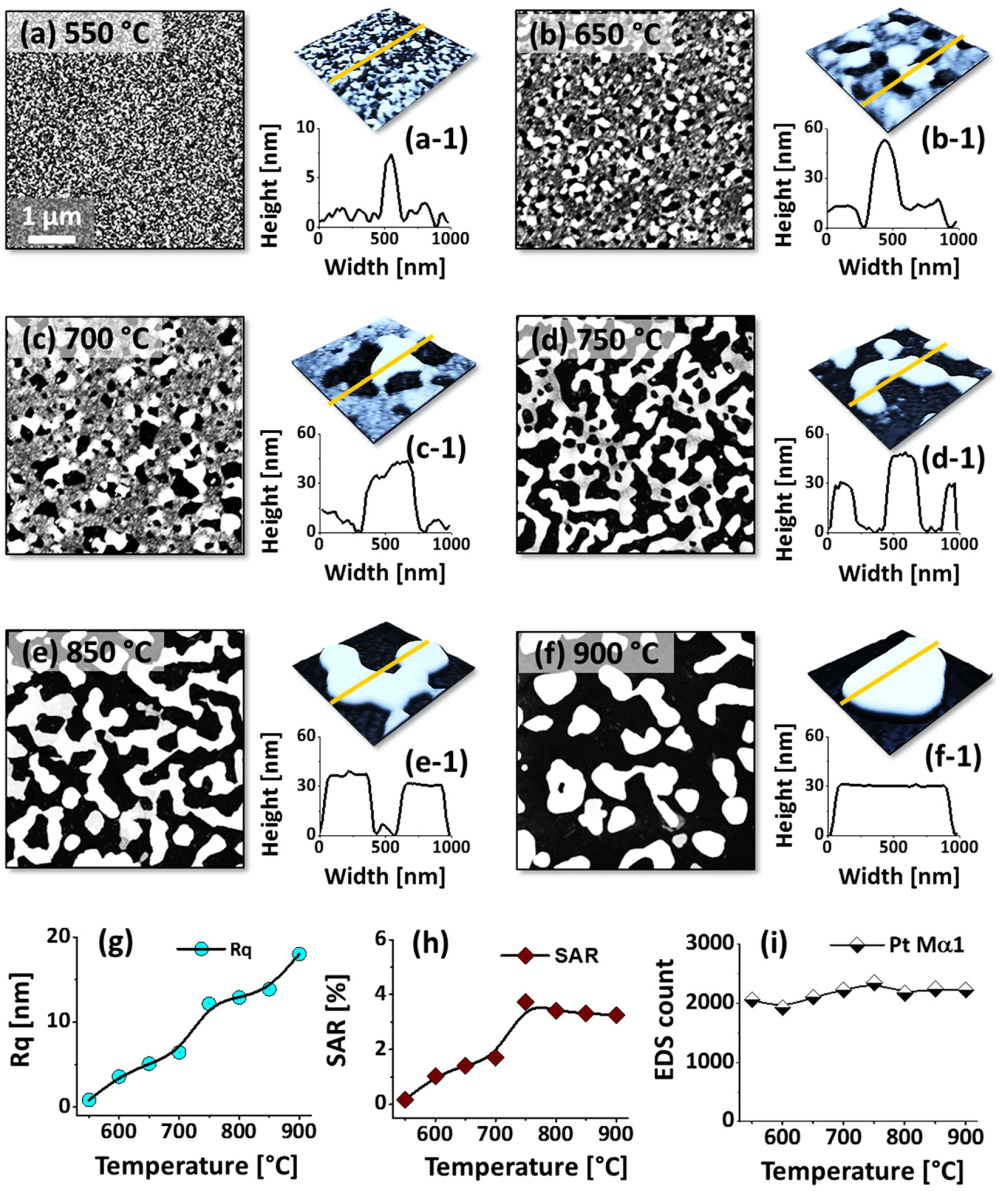
Formation of connected Pt NPs with the In_5nm_/Pt_15nm_ bilayer, annealed between 550 and 900 °C for 450 s. (**a**–**f**) AFM top-views of 5 × 5 μm^2^. (a-1)–(f-1) 3D-side views of 1 × 1 μm^2^ and cross-sectional line profiles. (**g**) Summary plot of Rq. (**h**) SAR plot. (i) EDS count summary of Pt Mα1 at 2.047 keV with respect to temperature.

Figure 8 shows the evolution of wiggly Pt nanoclusters on sapphire with the increased Pt layer thickness to 15 nm, 3 times higher, while keeping the same In layer thickness (5 nm) as in the first set. This set of samples was prepared to realize the Pt thickness effect on the evolution of nanostructures at a constant In thickness and growth conditions. At a first glance, the evolution of Pt nanostructures was drastically varied as compared to the previous cases. As displayed by the AFM top-views, various growth stages of Pt nanostructures were observed such as: void nucleation and growth, irregular-connected Pt nanoclusters formation and finally nanocluster breakdown resulting in the formation isolated irregular NPs. In this case, the overall thickness of In/Pt bilayer was similar to the previous set, however, the Pt content was increased in the bilayer, which resulted in the altered dewetting behavior. As the thickness of Pt layer was increased by three times at the identical In thickness, the stability against dewetting can also be increased correspondingly. On the other hand, the degree of interdiffusion with the Pt top-layer, In-Pt alloying and overall diffusivity of can also be hindered, which ultimately can result in the lower degree of dewetting at identical temperature. In specific, at 550 °C, the surface only became rougher by the formation tiny grains and voids as shown in Fig. 8(a). The height of small grains was normally ranged less than 3 nm, however, some grains were unexpectedly grown up to 8 nm as shown in Fig. 8(a-1), which can be related to the random anisotropic diffusion of atoms. By increasing the temperature, the surface diffusivity of metal atoms as well as the rate of In atom sublimation can also be increased. Consequently, at 650 °C, the dewetting process was further advanced by the void growth and coalescence with the neighboring ones due to the enhanced surface diffusion of atoms and sublimation of In, which is clearly observed in AFM top and side-views in Fig. 8(b),(b-1). A large portion of substrate area was exposed as the voids were mostly coalesced at 750 °C and irregular-connected (network-like) Pt nanoclusters with the average height of ~50 nm were formed. The void growth process can be driven by the interfacial energy minimization between the film and sapphire as discussed^27^. When the temperature was increased to 900 °C, the connected Pt nanoclusters were fragmented into the isolated NPs due to the surface energy anisotropy and Rayleigh-like instability of the cluster fringes^37–39^. For the isolated-irregular Pt NPs, they showed a compact configuration with the increased interparticle spacing, which can be the natural tendency to reduce the surface and interface energy^26^. The closer view of surface morphology evolution at each temperature is displayed by the corresponding AFM 3D side-views in Fig. 8(a-1)–(f-1). The cross-sectional line profiles extracted from the typical structures in AFM images show the dimension and shape of nanostructures. By a simple estimation, the vertical and lateral dimension of Pt NPs were increased by about 5 and 10 times respectively between 550 and 900 °C. In addition, the Rq and SAR analysis also suggest the gradual increment of corresponding values along with the evolution of Pt NPs. In case of SAR, it was slightly decreased above 750 °C, which can be due to the sharp reduction of average surface coverage of Pt nanoclusters. Furthermore, the EDS spectra analysis confirmed the presence of only Pt and substrate elements. The amount of In deposition was 3 times lower than the previous set and In was nearly completely sublimated as confirmed by the EDS spectra and summary plot in Fig. 8(i).

**Figure 9:**
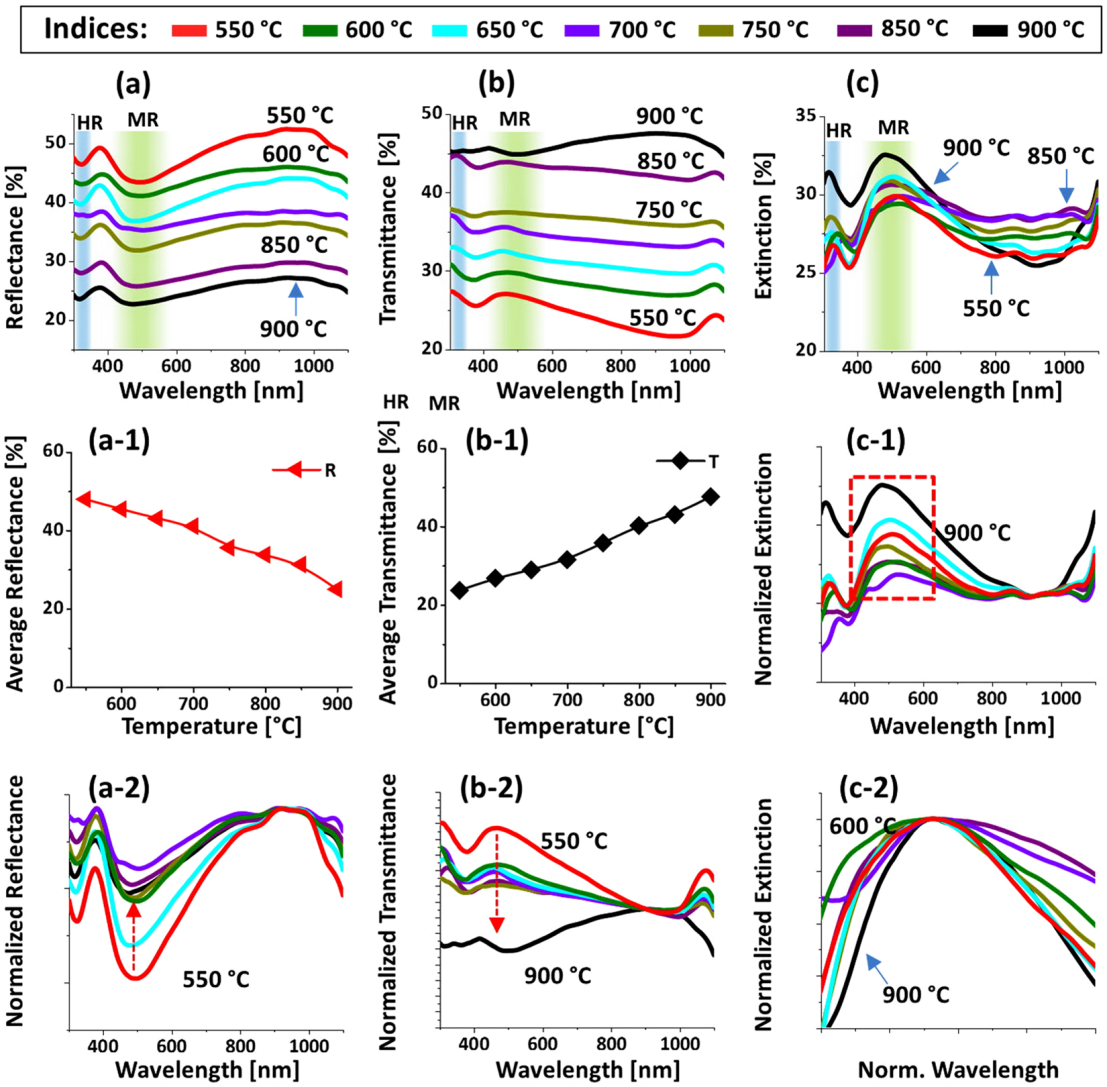
Optical properties of the Pt NPs fabricated with the In_15nm_/Pt_5nm_ bilayer between 550 and 900 °C for 450 s. (**a**) Reflectance (R) spectra. Formation of multipolar resonance (MR) and higher order resonance (HR) modes are indicated by color bands in (**a**–**c**). (a-1) Summary plot of R. (a-2) Normalized reflectance spectra. (**b**) Transmittance spectra. (b-1) Summary plot of T. (b-2) Normalized transmittance spectra. (**c**) Extinction spectra. (c-1) Normalized extinction spectra. (c-2) Enlarged extinction peaks between 400 and 700 nm, boxed region in (c-1).

Figure 9 shows the optical characterization of large and connected Pt nanoclusters by the reflectance, transmittance and extinction spectra. As a sharp contrast to the previous sets with small isolated and spherical Pt NPs, the optical properties in this sets were significantly varied. For instance, the reflectance spectra shown in Fig. 9(a) clearly demonstrates dips in the UV region at ~320 nm in the visible region at ~495 nm for all samples, which indicated the distinct optical behavior of grain-voids and large-connected nanoclusters as compared to the isolated-regular NPs in the previous cases. Since the Pt nanostructures are generally connected and larger with the average width of 500 nm and height of 30 nm, the excitation of various multipoles (dipoles, quadrupoles and higher order modes) can be expected by the interaction of photons^40^. The various multipoles (MR) can be superimposed leading to the formation of single resonance mode, which can be assigned to the visible region dip in the reflectance spectra^40^. The UV dip can be contributed to even higher order resonance modes as well as the inter-band transition of electrons^28,41^. The reflectance dips can be correlated to the enhanced absorption and/or forward scattering of photons due to the multipole resonance (MR)^29^. In which the quadrupolar resonance mode can be more significant as compared to other modes due to the larger size of Pt NPs^28,31^. The transmittance spectra in Fig. 9(b) generally showed shoulders in the UV (~320 nm) and in the visible region (~485 nm), also a distinction from the previous sets. These shoulders can be attributed to the pronounced forward scattering of light with the multipolar resonance mode of large Pt nanoclusters^41^. When the isolated Pt NPs were obtained at 900 °C, the transmittance behavior was readily shifted as it showed dip formation in the visible region in 9(b). As observed in previous sets, the absorption was enhanced in the visible region whereas forward scattering can be decreased for the case of isolated Pt NPs. The average reflectance and transmittance showed similar behavior as before with the surface coverage of Pt NPs as shown in Fig. 9(a-1),(b-1) respectively. The extinction spectra in Fig. 9(c) exhibited a narrow peak in the UV and a broader peak in the visible region corresponding to the multiple (MR) and higher order resonance (HR) mode of Pt nanoclusters^28^. As the Pt nanostructures were connected with a wide size distribution, the LSPR peaks were generally broad as compared to the previous sets. From the normalized reflectance in Fig. 9(a-2), it can be observed that the intensity of multipole resonance dip was gradually increased along with temperature, which can be correlated to the increased backward scattering with the evolution of Pt NPs with temperature. Similarly, the normalized transmittance spectra in Fig. 9(b-2) suggested the reduced forward scattering and increased absorption with the gradual isolation of Pt NPs from the widely connected nanoclusters. The overall LSPR effect was enhanced with the gradual dewetting of Pt NPs from high coverage and widely connected nanocluster as shown in Fig. 9(c-1), which suggests the improved absorption and scattering in the visible region. The formation of isolated NPs also influenced the LSPR peak band-width as the LSPR peaks became narrower as shown in Fig. 9(c-2)^30^.

## Conclusions

In summary, semi-spherical Pt NPs with the variety of size and spacing were demonstrated via the enhanced dewetting of Pt thin films using a sacrificial In layer. By comparing with the previous reports of intrinsic Pt film dewetting, a sharp contrast was demonstrated along with the introduction of sacrificial In layer, readily catalyzed the dewetting process. The In component was found to have a substantial impact on the dewetting process, which profoundly enhanced the overall diffusivity by means of interdiffusion and alloying with the Pt atoms. Along with the increased temperature, the In atoms were completely desorbed by the sublimation, which resulted in the formation of more isolated and well-developed Pt NPs. Various structural configurations, size and density of Pt NPs were demonstrated by simply changing the thickness of In and Pt in the bilayer at similar growth conditions. Results demonstrated the formation of regular and well-developed Pt NPs at relatively much low temperatures than the previous reports. Therefore, the high temperature processing of Pt film during the fabrication process can be adequately addressed by this approach, which can also provide an additional flexibility for Pt nanostructure fabrication. Furthermore, these Pt nanostructures demonstrated dynamic LSPR characteristics in the UV-visible region based on their configurations, size and spacing, i.e the global trend of enhanced absorption and narrowing of LSPR band along with the larger size and uniform shape of Pt NPs. Finally, these findings can be beneficial and scalable for the fabrication of Pt NPs as well as other metallic nanostructures for the plasmon mediated optical, electronic and catalytic systems.

## Methods

Both-side polished 430 μm-thick c-plane sapphire (0001) wafers with the ±0.1° off axis were utilized in this experiment. The sapphire offers low surface energy, high thermal and chemical stability and high inter-diffusion resistance. Furthermore, the transparency allows the direct measurement of transmittance, which helps to probe the optical response of NPs more effectively. Initially, the sapphire wafers were diced and degassed in a pulse laser deposition (PLD) chamber under 1 × 10^−4^ Torr at 600 °C for 30 min. The surface morphology, reflectance and transmittance spectra of bare sapphire are shown in Fig. S1. The degassed substrates were then used for the deposition of In/Pt bilayers and subsequent nanostructure fabrications. The deposition of In and Pt films was sequentially carried out in a sputter chamber using 99.999% purity targets of corresponding materials under 1 × 10^−1^ Torr. The deposition rate of 0.05 nm/s at 5 mA ionization current was utilized for both layers and the thickness was controlled by the duration of deposition. Three series of samples were prepared to experiment the alteration in the dewetting process and difference in the surface morphologies. In the first series, 5 nm of Pt layer was deposited on 5 nm of In layer, denoted as In_5nm_/Pt_5nm_ bilayer. Similarly, the second and third series of samples were prepared with the In_15nm_/Pt_5nm_ and In_5nm_/Pt_15nm_ bilayers respectively. The deposited sample was transferred to the PLD chamber for a subsequent annealing. The chamber pressure was lowered to below 1 × 10^−4^ Torr and the temperature was ramped up at 4 °C sec^−1^ to reach the target temperatures between 550 and 900 °C. The dwelling duration of 450 s was allocated to each sample at each constant target temperature. To insure the consistency, the overall annealing process was controlled by a computer recipe program. To terminate the growth, the heating system was turned off while keeping the chamber vacuum identical until the temperature was cooled down to an ambient in the course of time. The surface morphology of as-fabricated Pt nanostructures was characterized by an atomic force microscope (AFM, Park Systems, South Korea) and scanning electron microscope (SEM, CoXEM, South Korea). The AFM scanning were carried out under non-contact mode using the same batch of tips having radius of curvature <10 nm, force constant 40 nm^−1^ and resonant frequency ~300 kHz. The detailed structural and dimensional evolution of nanostructures are presented in terms of top-views, side-views, cross-sectional line profiles, roughness (Rq) and surface area ratio (SAR). The elemental analysis was carried out by an energy dispersive x-ray spectroscope (EDS, Thermo Fisher, Noran System 7, USA). The optical characteristics were acquired by an UNIRAM II system (UniNanoTech Co. Ltd, South Korea), equipped with the ANDOR sr-500i spectrograph (Oxford instruments, United Kingdom), CCD detector and various optics. Combined halogen and deuterium lamps (OCEAN optics, United Kingdom) were used as a light source to excite the samples, which covers the UV-VIS-NIR regimes of electromagnetic spectrum.

## Supplementary information

Supplementary Materials for Publication
