# Supplementary material for: Improved Configuration and LSPR Response of Platinum Nanoparticles via Enhanced Solid State Dewetting of In-Pt Bilayers
Source: Sci Rep. 2019 Feb 4;9:1329. doi: 10.1038/s41598-018-37849-0 (PMC6362192; doi:10.1038/s41598-018-37849-0)
Supplement: Supplementary file 1 — Supplementary Materials for Publication [file 41598_2018_37849_MOESM1_ESM.docx]

**Supplementary Materials
for Publication**

**Improved Configuration and LSPR Response of Platinum Nanoparticles via Enhanced Solid State Dewetting of In-Pt Bilayers**

Sundar Kunwar, Mao Sui, Puran Pandey, Zenan Gu, Sanchaya Pandit and Jihoon Lee^*^

Department of Electronic Engineering, College of Electronics and Information, Kwangwoon University, Nowon-gu Seoul 01897, South Korea.  *Correspondence e-mail: jihoonlee@kw.ac.kr

**
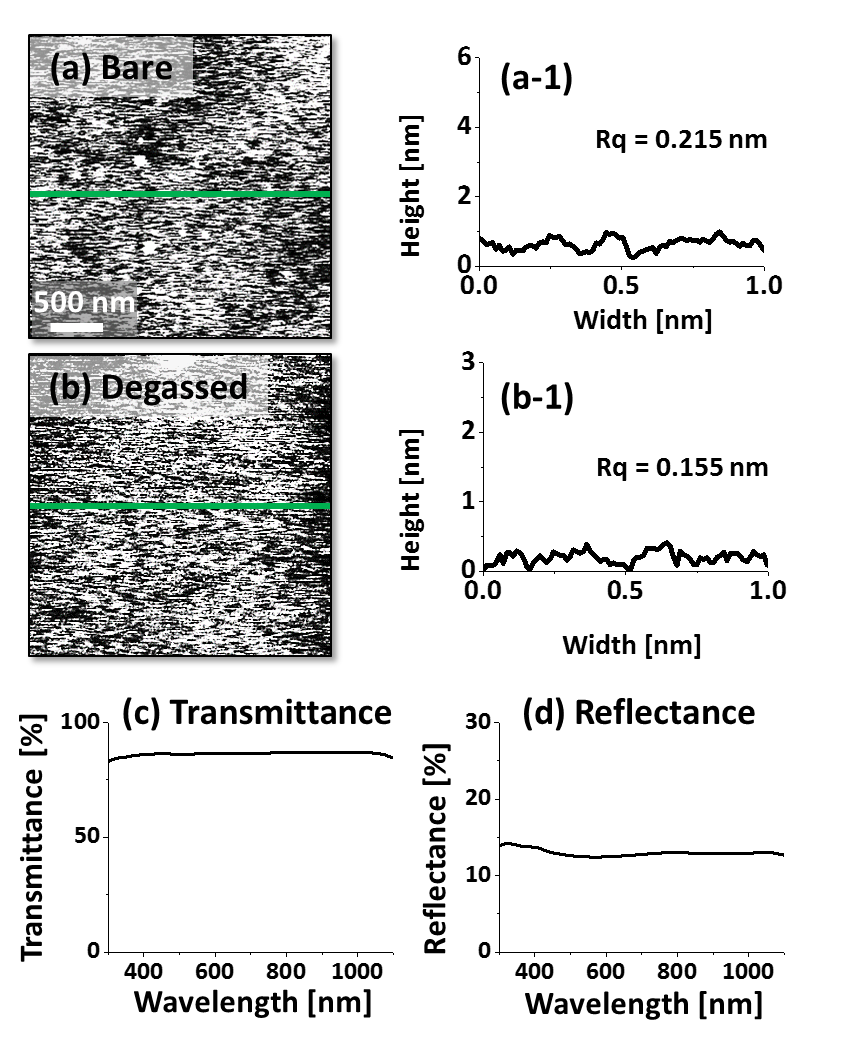
**

**Figure S1:** AFM top-views of bare sapphire (0001) (a) before and (b) after degassing. (a-1) - (b-1) Corresponding line profiles of the AFM images with RMS roughness (Rq) values. After degassing the surface becomes much cleaner and smoother as depicted by the line profiles and Rq values. (c) Transmittance spectra with ~ 86 % average transmittance within the wavelength between 300 and 1100 nm. (d) Reflectance spectra with ~ 13 % average reflectance within the same wavelength regime. The transmittance and reflectance exhibit uniform response within the wavelength.

**
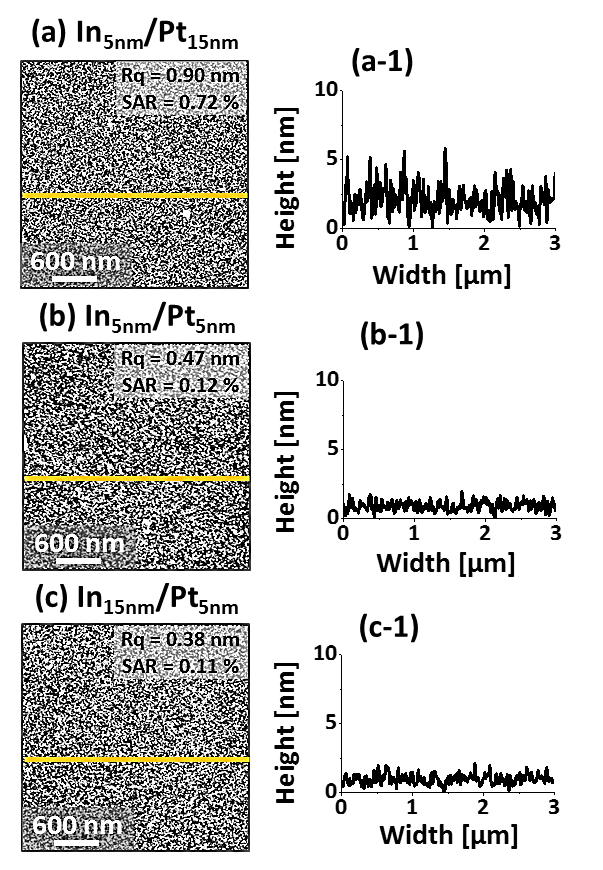
**

**Figure S2:** (a) – (c) AFM top-views of as-deposited In/Pt bilayers of various thickness as labelled prior to the annealing. The RMS roughness (Rq) and surface area ratio (SAR) are increased with the thickness of bilayer. (a-1) – (c-1) Cross-sectional line profiles extracted from the corresponding AFM top-views.


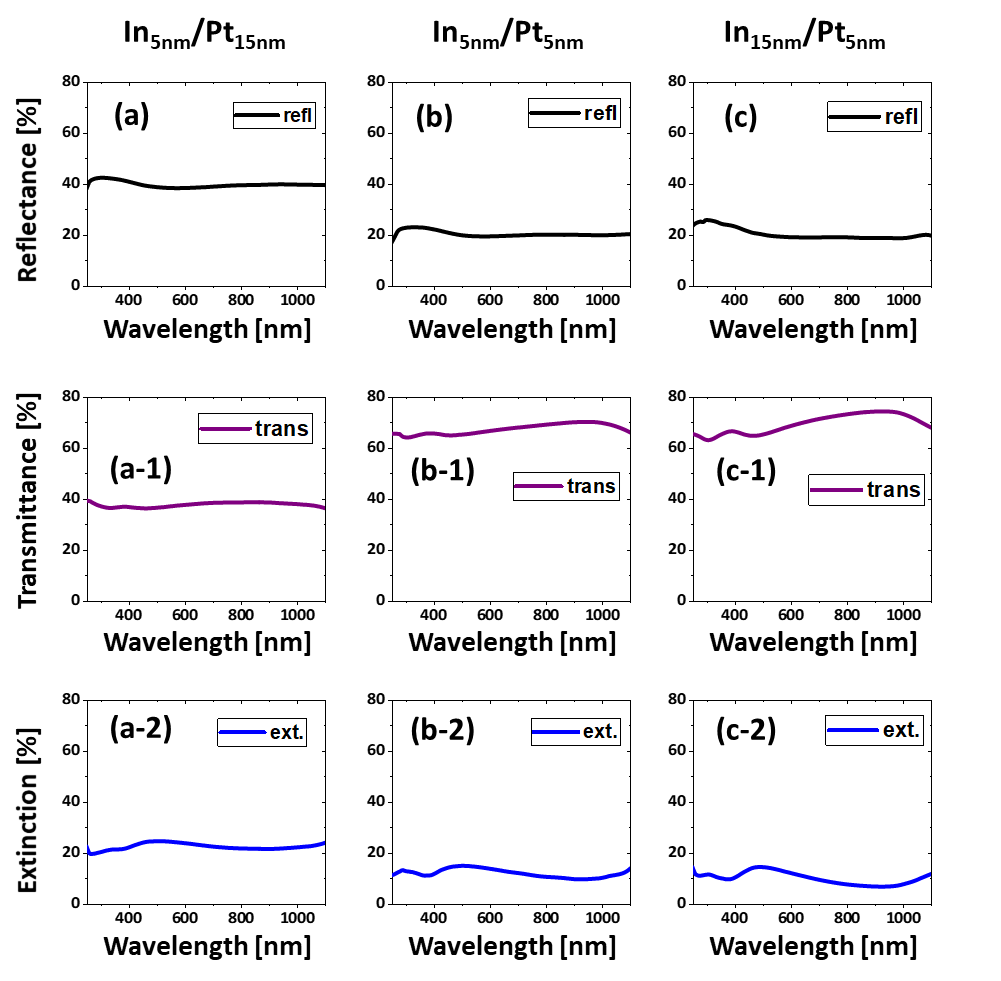


**Figure S3:** Optical analysis of the as-deposited In/Pt bilayers of various thickness as labelled. (a) – (b) Reflectance spectra. (a-1) – (c-1) Transmittance spectra. (a-2) – (c-2) Extinction spectra. The thin films generally demonstrated flat reflectacne spectra for all three different In/Pt bilayers. In the case of transmittance minor dips were realized in the UV and VIS regions, which can be correlated to the weak absorption by the deposited films. Similarly, the extinction spectra exhibits minor bumps in the UV and VIS region corresponding to the LSPR absorption dips. The peaks and dips can be correlated to the characteritic absorption dips of the In/Pt bilayers, which was largely enhaced after the evolution of definite NPs.

**
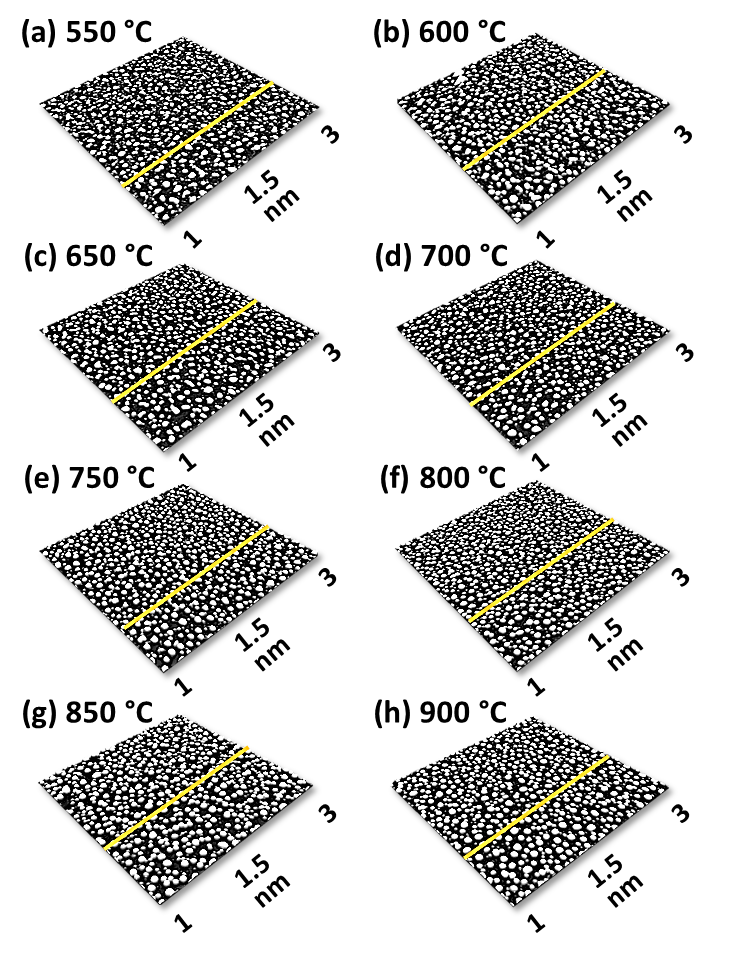
**

**Figure S4:** Evolution of Pt NPs with the In_5nm_/Pt_5nm_ bilayer based on the annealing at different temperautres between 550 and 900 °C for 450 s. (a) – (h) AFM side-views of 3 × 3 μm^2^.


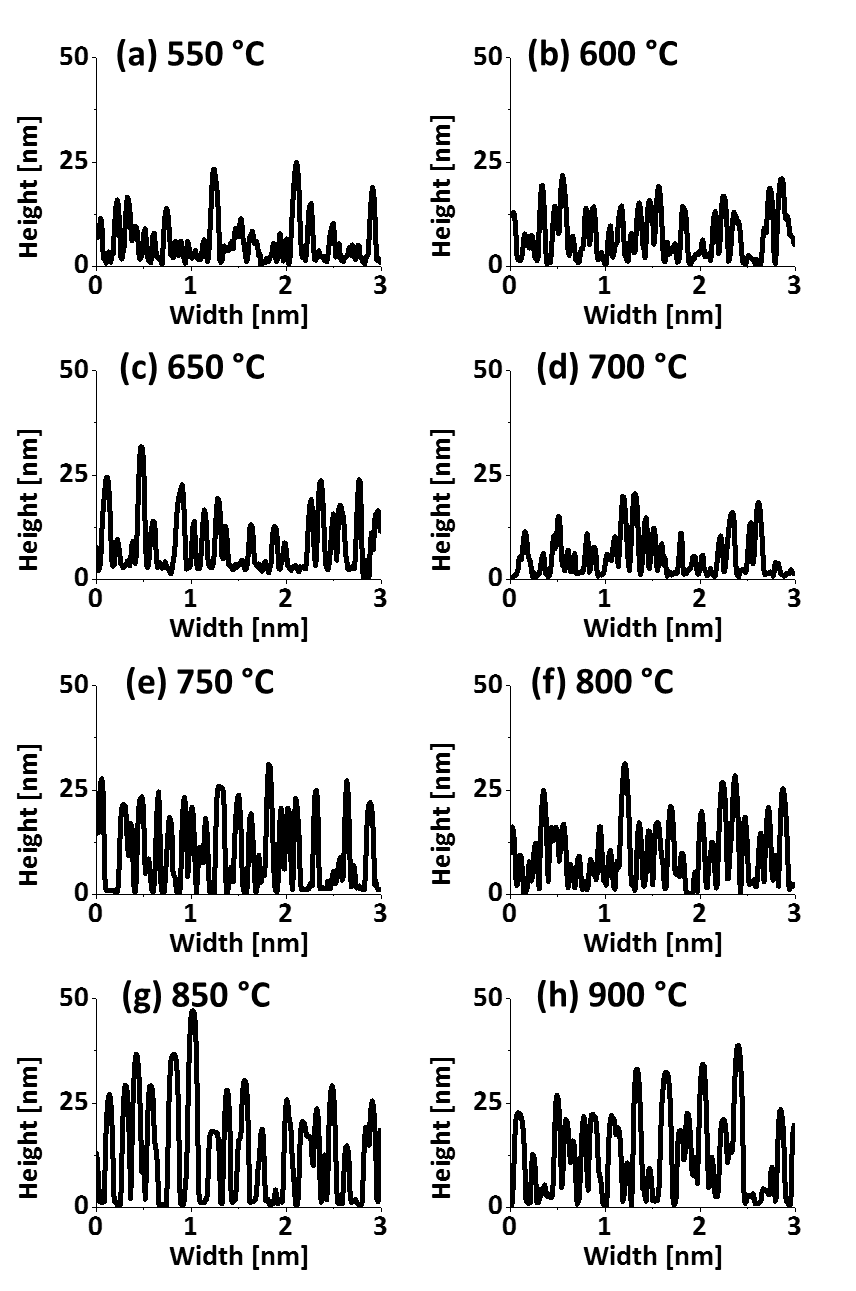


**Figure S5:** (a) – (h) Cross-sectional line profiles of Pt NPs extracted form the AFM side-views as shown in Fig. S3.


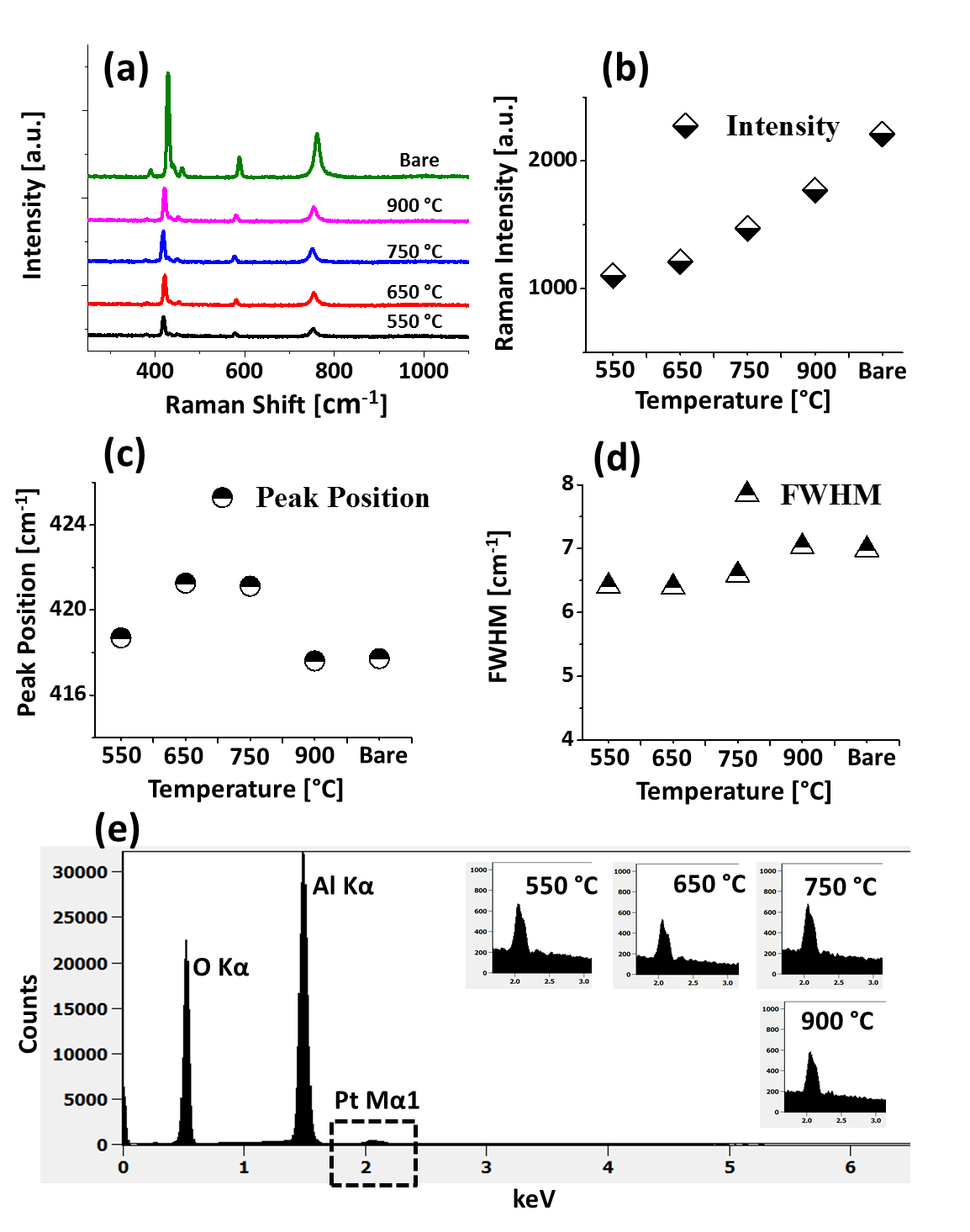


**Figure S6:** (a) Raman spectra of the Pt NPs on sapphire fabricated with In_5nm_/Pt_5nm_ bilayer between 550 and 900 °C. The Raman spectra were measured using 532 nm laser at 220 mW. Each sample exhibited six Raman bands namely the A_1g_ vibration mode at ~ 416.53 cm^-1^ and E_g_ vibration modes at ~ 378.24, 446.83, 575.78 and 749.65 cm^-1^ [1]. (b) – (d) Summary plots of the intensity, peak position and FWHM of A_1g_ with respect to the temperature. (e) EDS spectra and the Pt peaks in the insets for the samples at different temperature.

1. Porto, S. P. S., and R. S. Krishnan. "Raman effect of corundum." The Journal of Chemical Physics 47, no. 3 (1967): 1009-1012.

**
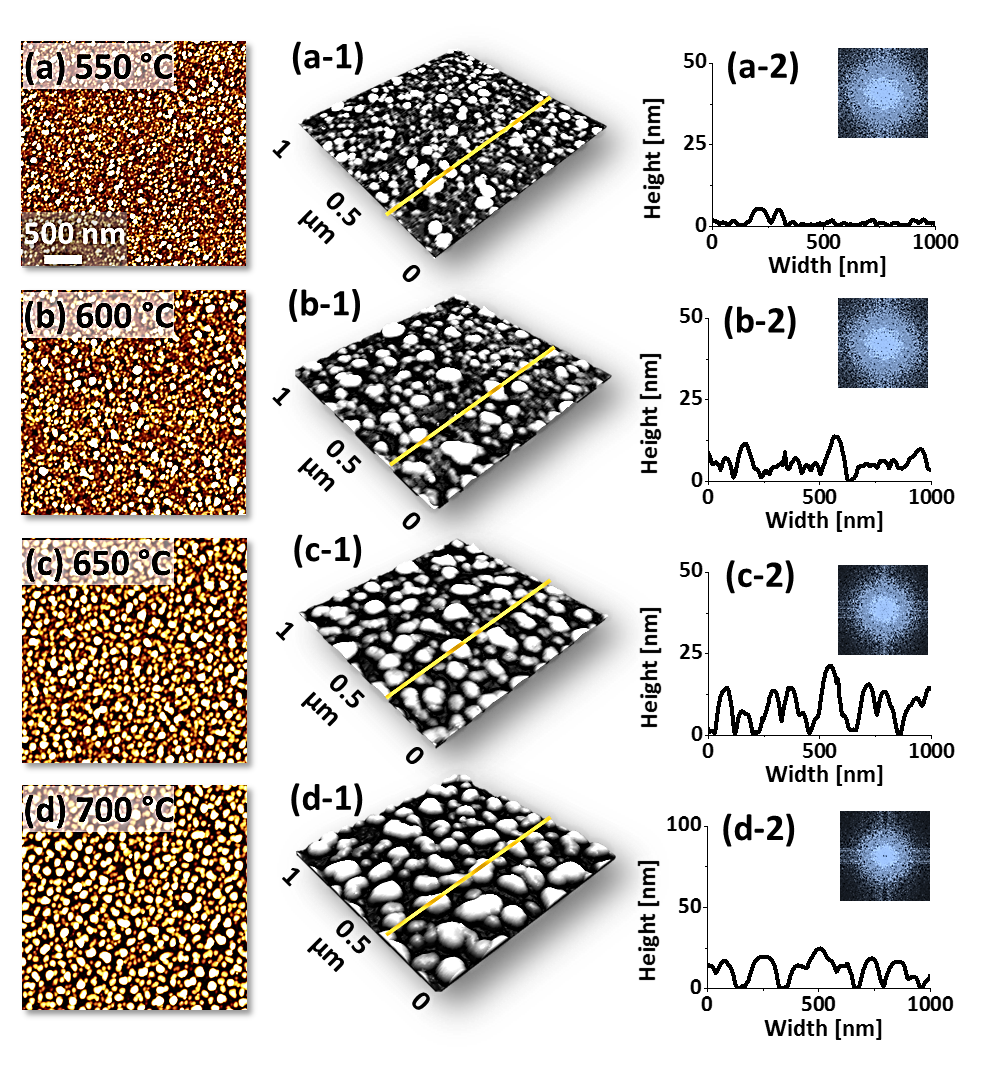
**

**Figure S7:** (a) – (d) Large scale AFM top-views of the Pt NPs fabricated by the annealing of In_15nm_/Pt_5nm_ bilayers between 550 and 700 °C for 450 s. (a-1) – (d-1) 3D side-views of 1 × 1 μm^2^. (a-2) – (d-2) Cross-sectional line profiles and Fourier filter transform (FFT) pattern of the AFM images. The FFT power pattern are the Fourier filter transform of each height pixel in AFM images. Larger FFT pattern represent the wide range of height distribution or poor uniformity and vice-versa.

**
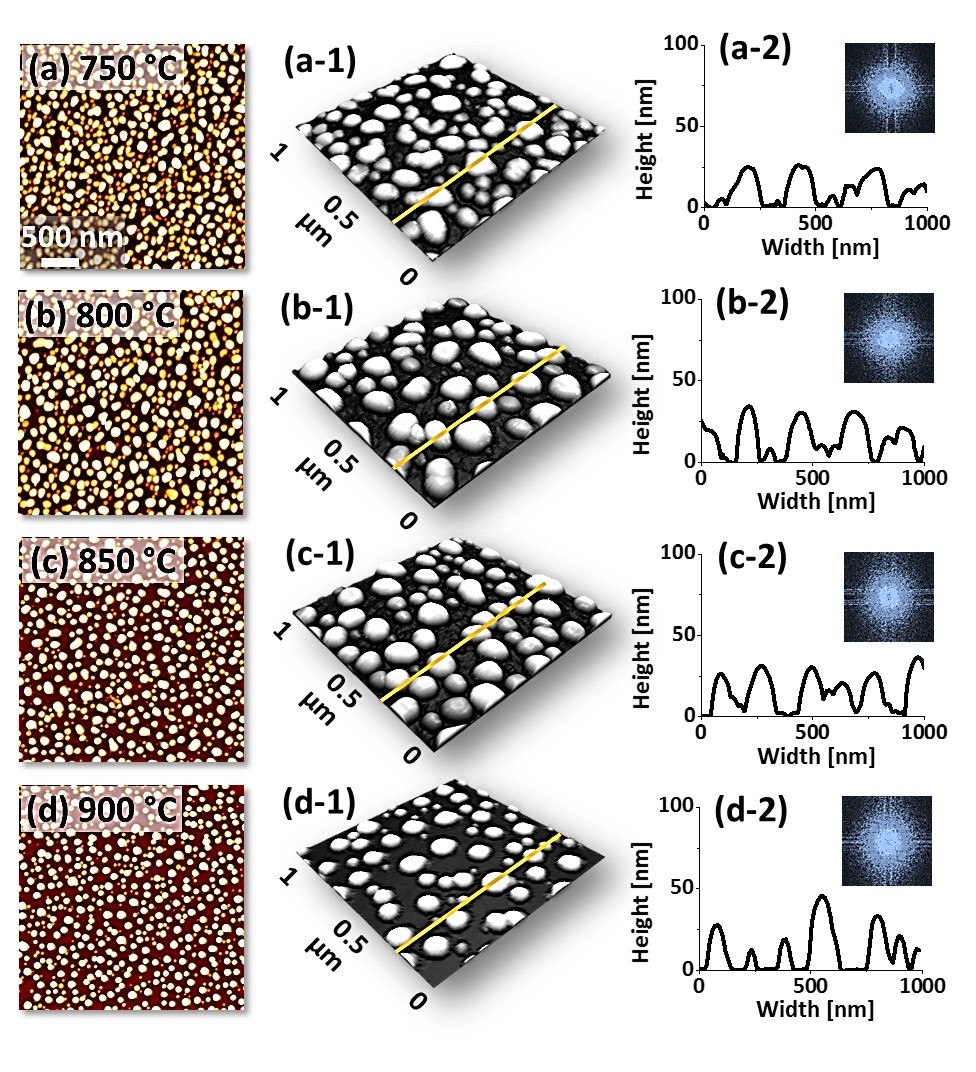
**

**Figure S8:** (a) – (d) Large scale AFM top-views of the Pt NPs fabricated by the annealing of In_15nm_/Pt_5nm_ bilayers from 750 to 900 °C. (a-1) – (d-1) 3D side-views of 1 × 1 μm^2^. (a-2) – (d-2) Cross-sectional line profiles and FFT pattern of the AFM images.

**
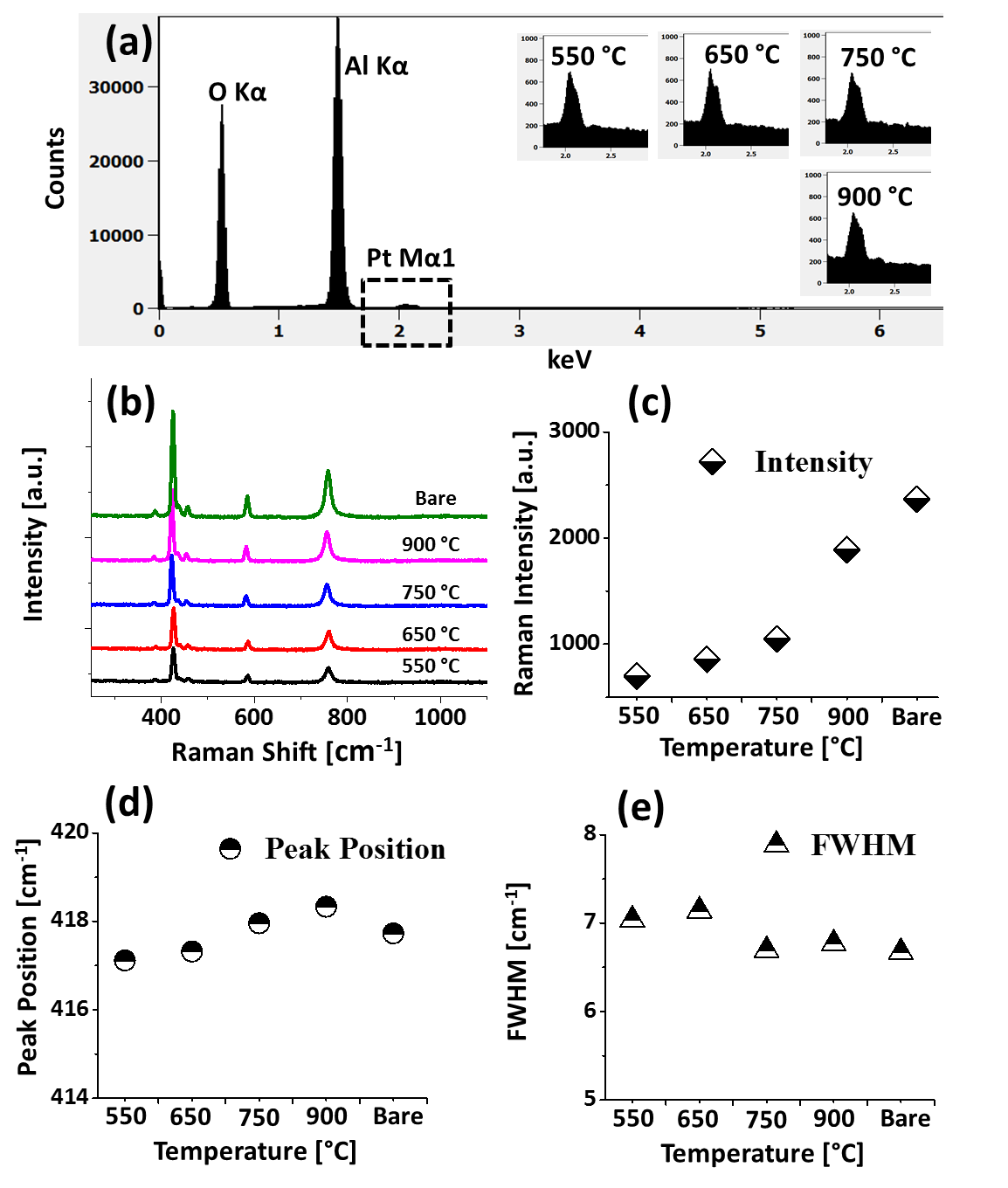
**

**Figure S9:** (a) EDS spectra of the Pt NPs on sapphire fabricated at various temperature as labelled with the In_15nm_/Pt_5nm_ bilayer. (b) Corresponding Raman spectra of the samples. (c) – (e) Summary plots of the intensity, peak position and FWHM of A_1g_ with respect to the temperature.

**
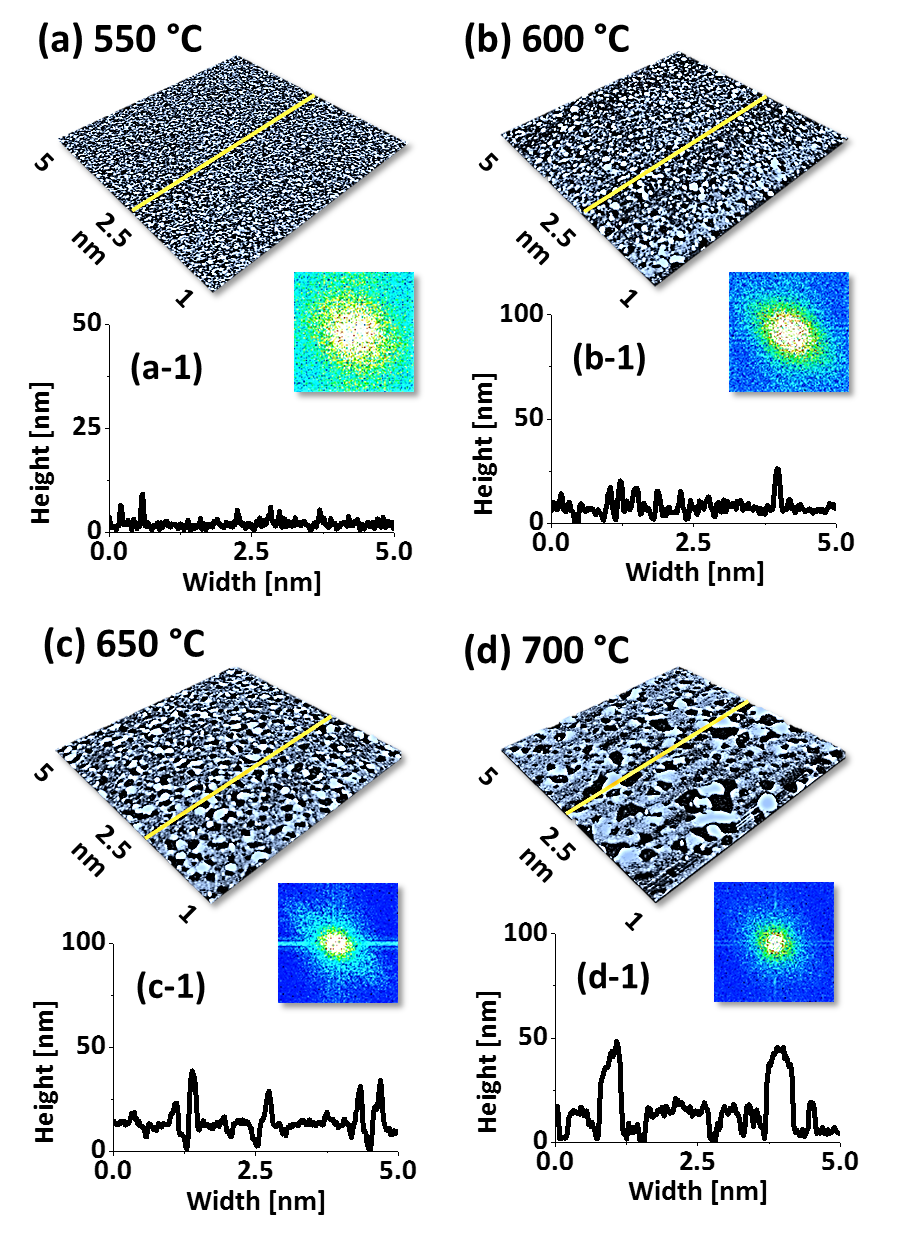
**

**Figure S10:** (a) – (d) Overview of the Pt NPs with large scale AFM images of the samples with the In_5nm_/Pt_15nm_ bilayers from 550 to 700 °C. (a-1) – (d-1) Corresponding Fourier filter transform (FFT) power spectra of the corresponding AFM images and line-profiles.


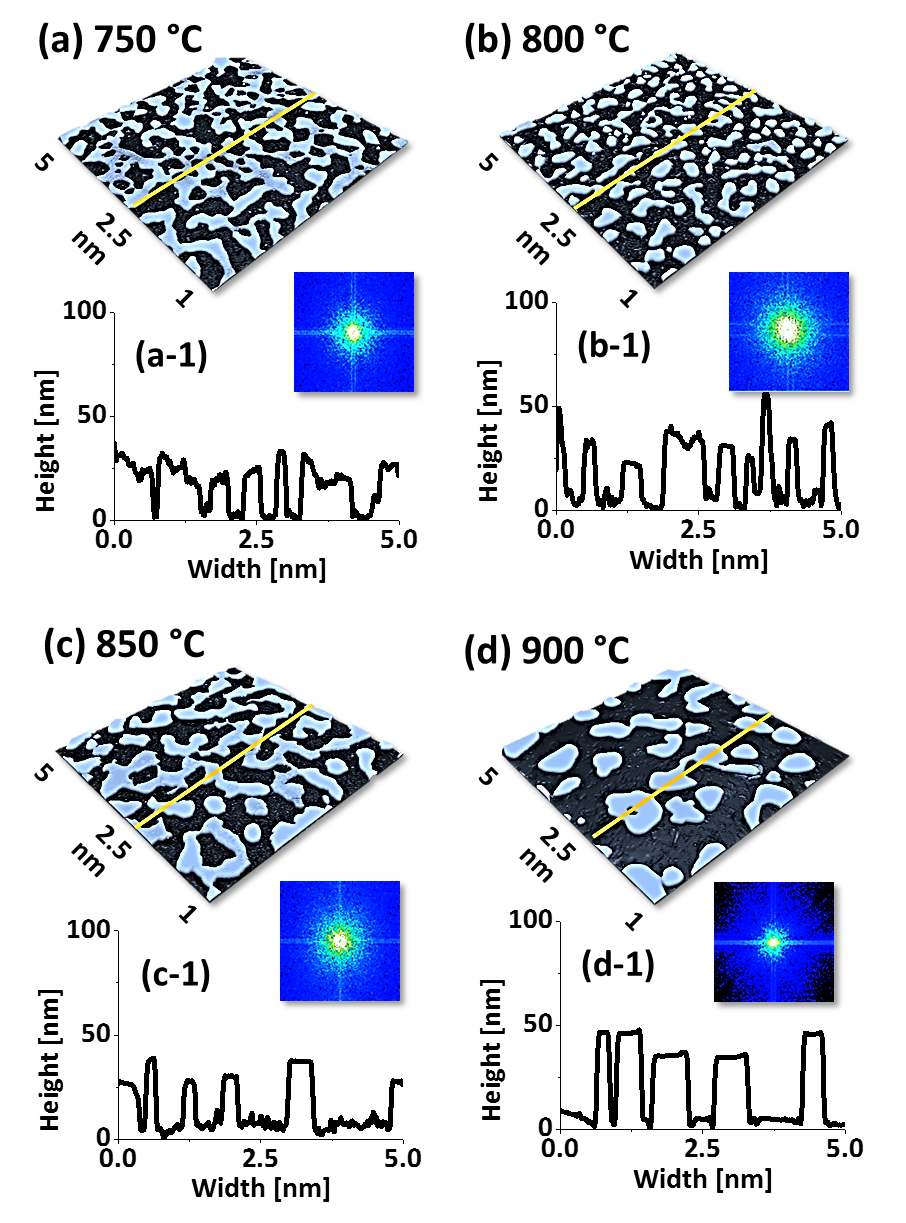


**Figure S11:** (a) – (d) Overview of the Pt NPs with large scale AFM images of the samples fabricated by the annealing In_5nm_/Pt_15nm_ bilayers from 750 to 900 °C. (a-1) – (d-1) Corresponding Fourier filter transform (FFT) power spectra of the corresponding AFM images and line-profiles.


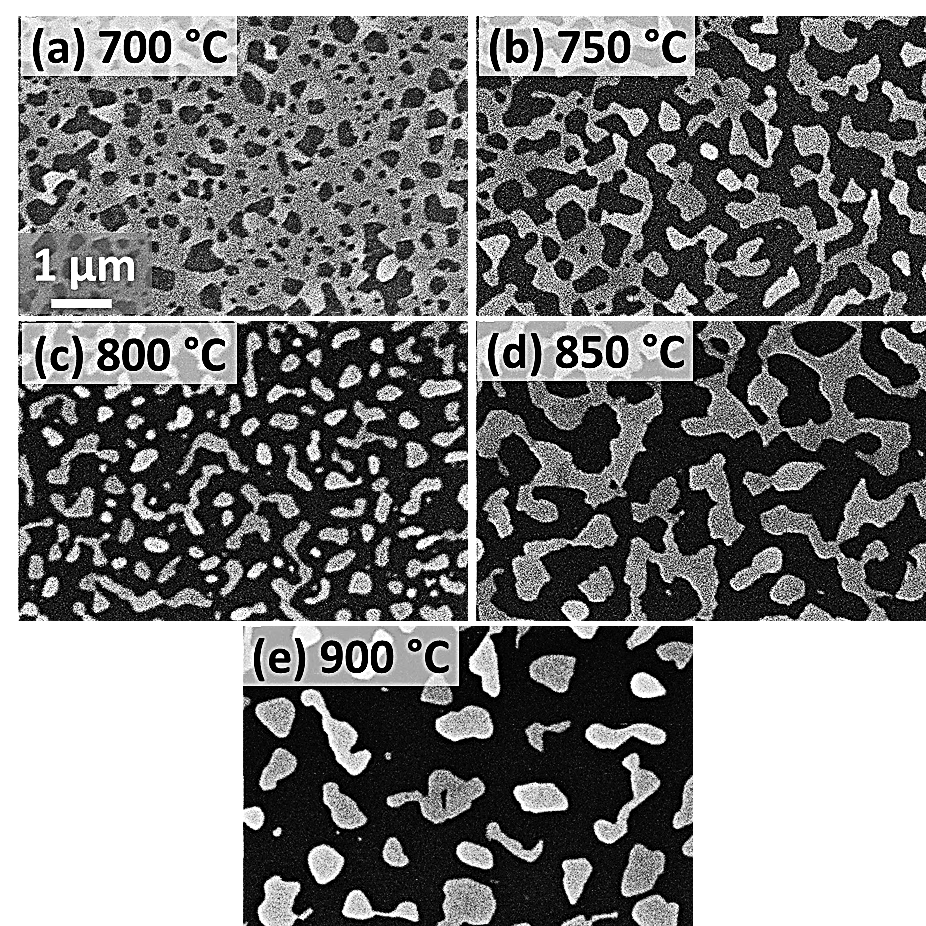


**Figure S12:** (a) – (e) Overview of the Pt NPs with large scale SEM images of the samples annealed from 700 to 900 °C for 450 s. The bilayer composition was In_5nm_/Pt_15nm_.

**
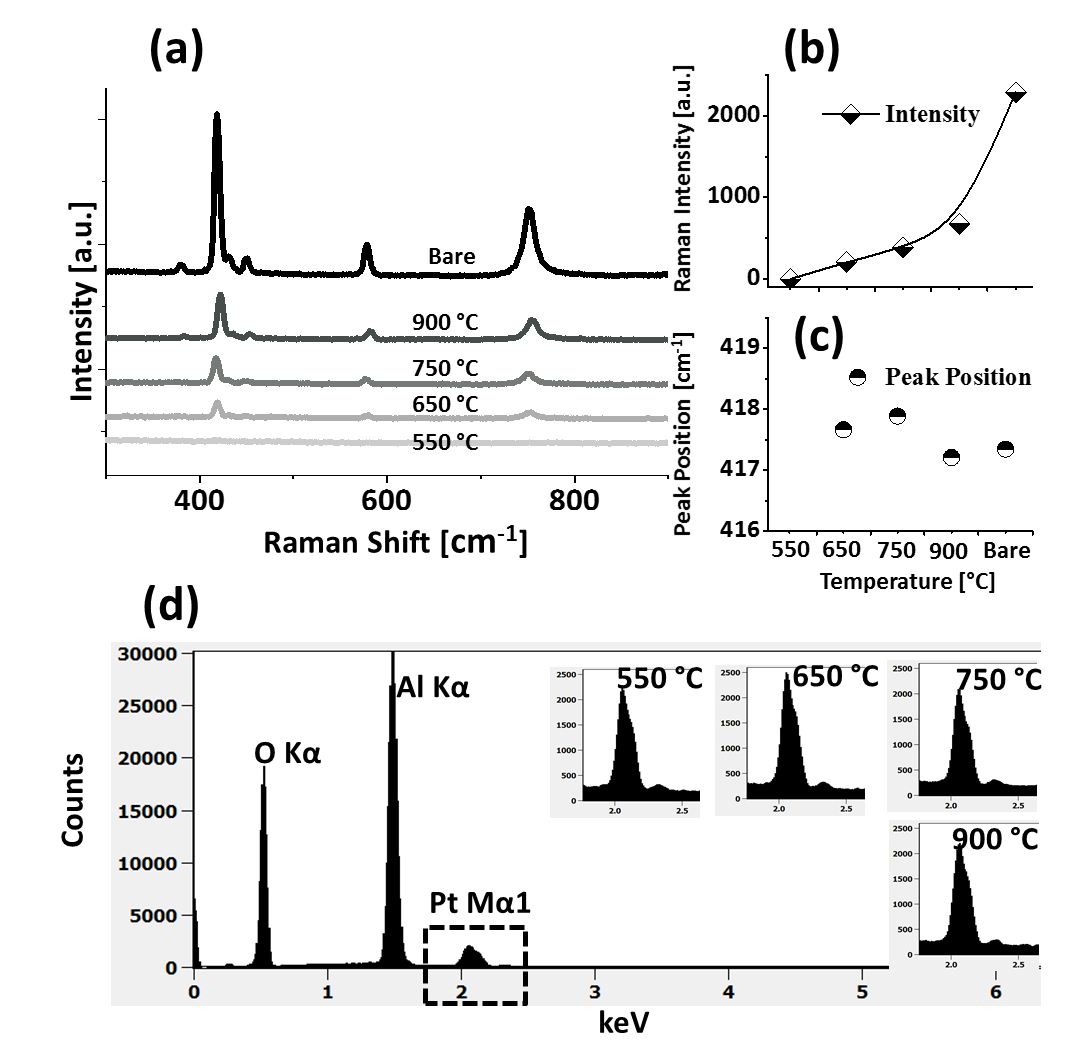
**

**Figure S13:** (a) Raman spectra of the samples at various temperature as labelled, the In_5nm_/Pt_15nm_ set. (b) Summary plot of the Raman intensity based on the A_1g_ vibration mode for various samples. (c) Summary plot of peak position shows slight right shift after fabrication of Pt NPs on sapphire. (d) EDS spectra of the samples depicting the O, Al and Pt peaks. The absence of In peak implies that the In has been completely sublimated before reaching the target temperature. In general, the Pt peak intensity of the samples is consistent throughout the temperature range as shown in the insets.

**Table S1:** Summary of RMS roughness (Rq) of the Pt NPs fabricated with the In/Pt bilayers at various annealing temperature. The Rq was generally increased along with the temperature due to increased size.

| **Temperature [°C]** | **Rq [nm]** | | |
| --- | --- | --- | --- |
|  | **In_5nm_/Pt_15nm_** | **In_5nm_/Pt_5nm_** | **In_15nm_/Pt_5nm_** |
| **550** | 0.838 | 4.985 | 1.033 |
| **600** | 3.58 | 5.670 | 2.790 |
| **650** | 5.102 | 5.905 | 5.067 |
| **700** | 6.440 | 6.650 | 6.550 |
| **750** | 12.147 | 8.735 | 7.105 |
| **800** | 12.90 | 8.750 | 9.30 |
| **850** | 13.86 | 8.958 | 9.890 |
| **900** | 18.007 | 8.758 | 10.350 |

**Table S2:** Summary of surface area ratio (SAR) of the Pt NPs fabricated at increasing annealing temperature using In/Pt bilayers of various compositions.

| **Temperature**  **[°C]** | **SAR [%]** | | |
| --- | --- | --- | --- |
|  | **In_5nm_/Pt_15nm_** | **In_5nm_/Pt_5nm_** | **In_15nm_/Pt_5nm_** |
| **550** | 0.175 | 4.433 | 0.555 |
| **600** | 1.027 | 4.633 | 1.408 |
| **650** | 1.419 | 5.662 | 4.698 |
| **700** | 1.711 | 6.241 | 4959 |
| **750** | 3.730 | 10.310 | 7.382 |
| **800** | 3.410 | 10.465 | 8.938 |
| **850** | 3.212 | 10.790 | 9.289 |
| **900** | 3.312 | 10.788 | 10.101 |

**Table S3:** Summary of average reflectance (R), transmittance (T) and extinction (E) of the Pt NPs fabricated at increasing annealing temperature using In_5nm_/Pt_15nm_ bilayers of various compositions.

| **Temperature [°C]** | **Composition** | | |  |
| --- | --- | --- | --- | --- |
|  | **In_5nm_/Pt_15nm_** | | | |
|  | **R [%]** | **T [%]** | **E [%]** |  |
| **550** | 47.879 | 23.593 | - |  |
| **600** | 45.347 | 26.716 | - |  |
| **650** | 42.977 | 28.835 | - |  |
| **700** | 40.993 | 31.434 | - |  |
| **750** | 35.484 | 35.722 | - |  |
| **800** | 33.690 | 40.177 | - |  |
| **850** | 31.205 | 42.973 | - |  |
| **900** | 24.860 | 47.547 | - |  |

**Table S4:** Summary of average reflectance (R), transmittance (T) and extinction (E) of the Pt NPs fabricated at increasing annealing temperature using In_5nm_/Pt_5nm_ bilayers of various compositions.

| **Temperature**  **[°C]** | **Composition** | | |
| --- | --- | --- | --- |
|  | **In_5nm_/Pt_5nm_** | | |
|  | **R [%]** | **T [%]** | **E [%]** |
| **550** | 26.604 | 55.348 | - |
| **600** | 25.306 | 57.809 | - |
| **650** | 24.630 | 58.744 | - |
| **700** | 23.869 | 60.567 | - |
| **750** | 22.696 | 64.609 | - |
| **800** | 21.439 | 67.263 | - |
| **850** | 19.830 | 71.033 | - |
| **900** | 18.531 | 73.873 | - |

**Table S5:** Summary of average reflectance (R), transmittance (T) and extinction (E) of the Pt NPs fabricated at increasing annealing temperature using In_15nm_/Pt_5nm_ bilayers of various compositions.

| **Temperature**  **[°C]** | **Composition** | | |
| --- | --- | --- | --- |
|  | **In_15nm_/Pt_5nm_** | | |
|  | **R [%]** | **T [%]** | **E [%]** |
| **550** | 26.67265 | 45.94017 | - |
| **600** | 24.86527 | 48.58673 | - |
| **650** | 23.63175 | 52.98921 | - |
| **700** | 22.42893 | 55.75079 | - |
| **750** | 20.98112 | 58.00242 | - |
| **800** | 19.13137 | 63.56923 | - |
| **850** | 17.30644 | 65.51365 | - |
| **900** | 12.88668 | 67.9063 | - |

**Table S6:** Summary of Raman intensity and peak position of the Pt NPs fabricated at increasing annealing temperature using In/Pt bilayers of various compositions.

| **Temperature [°C]** | **Composition** | | | | | | |
| --- | --- | --- | --- | --- | --- | --- | --- |
|  | **In_5nm_/Pt_15nm_** | | | **In_5nm_/Pt_5nm_** | | **In_15nm_/Pt_5nm_** | |
|  | **Intensity** | **Peak**  **Position** | **Intensity** | | **Peak**  **Position** | **Intensity** | **Peak**  **Position** |
| **550** | 0 | 0 | 1100.97 | | 418.68 | 698.56 | 417.14 |
| **650** | 207.57 | 417.66 | 1212.19 | | 421.26 | 857.60 | 417.32 |
| **750** | 385.63 | 417.88 | 1471.30 | | 421.11 | 1049.06 | 417.96 |
| **900** | 674.77 | 417.21 | 2210.54 | | 417.71 | 1889.81 | 418.33 |
